# Assessment of a Functional Yogurt Enriched with Anthocyanin-Loaded Nanoliposomes: Sensory Evaluation and Physicochemical Stability During Cold Storage

**DOI:** 10.3390/ijms26199637

**Published:** 2025-10-02

**Authors:** Miguel Ángel Robles-García, Carmen Lizette Del-Toro-Sánchez, Linthia Jovana Tapia-Beiza, Melesio Gutiérrez-Lomelí, María Guadalupe Avila-Novoa, Ariadna Thalía Bernal-Mercado, Francisco Javier Reynoso-Marín, Fridha Viridiana Villalpando-Vargas, Alejandra Vázquez-Aguilar, Ernesto Ramírez-Briones, Ricardo Iván González-Vega

**Affiliations:** 1 Department of Medical and Life Sciences, Cienega University Center (CUCIÉNEGA), University of Guadalajara, Av. Universidad 1115, Lindavista, Ocotlán 47820, Jalisco, Mexico; miguel.robles@academicos.udg.mx (M.Á.R.-G.); melesio.gutierrez@academicos.udg.mx (M.G.-L.); maria.anovoa@academicos.udg.mx (M.G.A.-N.);; 2 Department of Research and Postgraduate in Food, University of Sonora, Blvd. Luis Encinas y Rosales S/N, Col. Centro, Hermosillo 83000, Sonora, Mexico; 3 Department of Health Sciences, University Center of the Valleys (CUVALLE), University of Guadalajara, Carr. a Guadalajara Km. 45.5, Ameca 46600, Jalisco, Mexico; linthia.tapia9419@alumnos.udg.mx (L.J.T.-B.);; 4 Department of Nanotechnology Engineering, University of La Ciénega of the State of Michoacán of Ocampo (UCEMICH), Avenida Universidad 3000, Lomas de la Universidad Neighborhood, Sahuayo 59103, Michoacán, Mexico; fjreynoso@ucemich.edu.mx; 5 Department of Cellular and Molecular Biology, University Center for Biological and Agricultural Sciences (CUCBA), University of Guadalajara, Periférico Norte N° 799 Núcleo Universitario, C. Prol. Belenes, Zapopan 45100, Jalisco, Mexico; 6 Department of Applied Ecology, University Center for Biological and Agricultural Sciences (CUCBA), University of Guadalajara, Periférico Norte N° 799 Núcleo Universitario, C. Prol. Belenes, Zapopan 45100, Jalisco, Mexico

**Keywords:** anthocyanins, nanoliposomes, potentially functional food

## Abstract

In the development of functional foods with therapeutic value, nanoliposomal carriers offer a promising strategy for enhancing the stability and efficacy of bioactive compounds in dairy matrices. This study evaluated the sensory acceptance and physicochemical stability of yogurt enriched with anthocyanin-loaded nanoliposomes during 21 days of refrigerated storage, assessing the impact of nanoencapsulation on compound preservation and quality. Nanoliposomes were synthesized using ultrasonic film dispersion and characterized for antioxidant and erythroprotective activities. Antioxidant capacity was assessed through DPPH, ABTS, and FRAP assays, while erythroprotective effects were evaluated via oxidative hemolysis using human erythrocytes of different ABO/RhD phenotypes. These were incorporated into artisanal yogurt, followed by physicochemical, microbiological, rheological, and sensory analyses. Anthocyanins showed strong antioxidant capacity, especially in ABTS (93.24%), DPPH (21.34%), and FRAP (1023.24 µM TE/g D.W.), reflecting their radical scavenging and reducing power. They also exhibited high erythroprotective activity, with greater antihemolytic effects in O RhD− blood and enhanced photoprotection against UVA in O RhD+ blood. Yogurt enriched with nanoliposomes showed improved color stability, reduced syneresis, and favorable rheological and sensory characteristics. These findings support nanoliposomes as molecular delivery systems in functional dairy matrices with potential nutraceutical applications targeting oxidative stress. Further work should explore molecular mechanisms and validate health-promoting effects.

## 1. Introduction

Lipid-based nanostructured delivery systems, particularly nanoliposomes, have emerged as efficient and versatile tools for the controlled release of bioactive and nutraceutical compounds [1]. Owing to their bilayer lipid membrane architecture, nanoliposomes can encapsulate both hydrophilic and lipophilic substances, protecting them from destabilizing environmental factors while enhancing stability, bioaccessibility, and bioavailability [2,3]. This approach has gained increasing attention in the food sector. Incorporating nanostructured systems into edible matrices not only preserves the functional integrity of bioactive ingredients during processing and storage but also modulates their release under physiological conditions [2,4,5]. Thus, nanoliposomes represent an innovative strategy for developing next-generation functional foods with enhanced therapeutic potential, particularly for the prevention of chronic diseases associated with oxidative stress and inflammation. Within this framework, the present study explores the development and stability of a functional yogurt enriched with loaded nanoliposomes, with emphasis on its physicochemical and sensory properties during cold storage [6,7,8]. Anthocyanins are potent antioxidant flavonoids but exhibit limited stability when exposed to light, pH fluctuations, and temperature variations. Nanoencapsulation in nanoliposomes provides a technological solution to improve their performance and integration into dairy matrices. Açaí anthocyanins, particularly cyanidin-3-glucoside and cyanidin-3-rutinoside, together with phenolic acids and flavonoids, contribute to its strong antioxidant capacity [9,10].

Interest in functional foods that confer health benefits beyond basic nutrition is growing, especially in the field of neuronutrition [11,12]. Bioactive compounds such as antioxidants, omega-3 fatty acids, vitamins, and polyphenols support central nervous system health by modulating inflammation, reducing oxidative stress, and enhancing neuronal plasticity—key mechanisms for preventing neurodegenerative and chronic diseases. Recent evidence has also linked ABO and RhD blood groups with disease susceptibility, suggesting that genetic predisposition and nutrient metabolism jointly influence health outcomes. Compounds such as fucoxanthin, β-carotene, gallic acid, quercetin, and ascorbic acid have demonstrated antioxidant and erythroprotective effects dependent on blood group, highlighting the value of personalized nutrition [12,13]. Although anthocyanins possess neuroprotective and anti-inflammatory potential, their instability under environmental stress limits their application. Encapsulation in nanoliposomes enhances their stability, enables controlled release, and protects them from degradation. Incorporating nanoliposome-encapsulated anthocyanins into foods such as yogurt may therefore improve both shelf life and health benefits, particularly for genetically susceptible individuals [1,14].

Previous studies have demonstrated that nanoencapsulation can improve the stability and controlled release of bioactive compounds in food matrices, such as fish oil [1,15]. However, the integration of anthocyanins into yogurt remains challenging due to their susceptibility to degradation during processing and storage, along with their limited bioavailability. In the absence of protective carriers, anthocyanins undergo rapid degradation through several mechanisms, including structural transformations such as hydration of the flavylium cation, oxidation, and cleavage of glycosidic bonds, which lead to color loss, reduced antioxidant activity, and diminished functional properties. To overcome these limitations, nanoliposomes have been investigated as promising carriers, offering protection against environmental degradation, improved bioavailability, and controlled release [6,14]. Nanoliposomes offer a promising solution by protecting against environmental degradation, enhancing bioavailability, and enabling controlled release. For example, the incorporation of anthocyanin-rich red elderberry extracts (A-RBE) into yogurt has been shown to reduce syneresis while increasing polyphenol content, anthocyanins, and antioxidant activity during digestion, without compromising probiotic viability [16,17,18]. Similarly, Robles-García et al. [19] reported that fucoxanthin-loaded nanoliposomes, produced via a scalable ultrasonic film dispersion method, exhibited high encapsulation efficiency and provided antioxidant stability during cold storage when incorporated into yogurt. Although nanoliposomes slightly reduced yogurt viscosity and firmness, they improved pH stability, titratable acidity, and water-holding capacity, thereby enhancing overall product quality. Notably, formulations containing 10% fucoxanthin nanoliposomes not only improved antioxidant capacity but also enhanced sensory attributes, indicating greater consumer acceptance. These findings highlight the potential of nanoliposomal delivery systems to improve the functionality and stability of dairy products, though further optimization of textural properties is still needed [20,21].

Although several studies have investigated the incorporation of anthocyanins or nanoliposome-encapsulated bioactives into dairy systems, including yogurt, there is limited evidence specifically addressing the use of açaí anthocyanin-loaded nanoliposomes in artisanal yogurt as effective carriers with potential antioxidant and erythroprotective effects against oxidative damage [22,23]. The aim of this study was therefore to evaluate the impact of incorporating anthocyanin-loaded nanoliposomes on key parameters such as the texture, acidity, syneresis, and sensory acceptance of yogurt formulations over time.

## 2. Results and Discussion

### 2.1. Biological Activity

#### 2.1.1. Antioxidant Activity

The antioxidant activities of anthocyanins were measured using three different assays: ABTS^+^•, DPPH•, and FRAP. Comparing the results of the ABTS and DPPH assays reveals a substantial difference in the antioxidant activity values. The ABTS assay yielded a significantly higher antioxidant activity value (93.24 ± 1.87%) compared to the DPPH assay (21.34 ± 2.45%). This variation could be attributed to differences in the mechanisms and reactivities of the radicals involved in each assay. The ABTS assay measures both hydrophilic and lipophilic antioxidants, while the DPPH assay primarily evaluates the ability of hydrophilic antioxidants to scavenge free radicals. Therefore, the higher antioxidant activity observed in the ABTS assay suggests that the anthocyanins may possess a greater capacity to scavenge ABTS radicals compared to DPPH radicals.

Regarding the FRAP assay, the antioxidant activity of anthocyanins is represented by a value of 1023.24 ± 51.25 µM TE/g D.W. The FRAP assay measures the ability of antioxidants to reduce ferric ions, indicating their reducing power, but does not indicate that the compound has the capacity to scavenge free radicals. The observed FRAP value suggests that anthocyanins exhibit a high capacity to donate electrons and reduce ferric ions, which is consistent with their antioxidant properties. The antioxidant activity of anthocyanins, as assessed by the ABTS, DPPH, and FRAP assays, demonstrates their potent radical scavenging and reducing properties. The variations in the antioxidant activity values obtained from different assays highlight the importance of employing multiple methods to evaluate the antioxidant capacity of anthocyanins comprehensively [24].

Anthocyanins are potent natural antioxidants that act as electron or hydrogen donors, effectively neutralizing free radicals such as ABTS and DPPH [25]. This neutralization results in measurable color changes, which are proportional to the antioxidant activity of the sample. A higher percentage of inhibition in both assays indicates a stronger radical scavenging capacity. In the ABTS assay, anthocyanins donate electrons to neutralize ABTS radicals, while in the DPPH assay, they convert DPPH radicals into stable, colorless DPPH-H molecules [25,26]. These widely used assays demonstrate the ability of anthocyanins to counteract oxidative stress through radical scavenging. In addition, the FRAP assay evaluates the reducing power of antioxidants; anthocyanins reduce ferric (Fe^3+^) to ferrous (Fe^2+^) ions, confirming their electron-donating capacity. Together, these assays highlight the antioxidant strength of anthocyanins and their potential health benefits [24,25,26].

Belonging to the flavonoid group, anthocyanins stand out among polyphenols due to their unique chemical structure, characterized by conjugated double bonds and multiple hydroxyl groups. This structure enhances their ability to stabilize free radicals and mitigate oxidative stress. Compared to other polyphenols and even certain vitamins, such as vitamin C and vitamin E, anthocyanins often exhibit superior antioxidant activity. Moreover, they can scavenge a broader range of reactive species and act synergistically with vitamins, contributing to enhanced cellular protection. Given their exceptional radical scavenging and reducing properties, anthocyanins are considered valuable bioactive compounds for human health. Their inclusion in the diet, through anthocyanin-rich foods like berries, grapes, and purple vegetables, can support the prevention of oxidative stress-related diseases. Overall, the evidence supports anthocyanins as effective functional ingredients in food systems, with promising implications for both nutritional and therapeutic applications [27,28].

#### 2.1.2. Erythroprotector Potential

The antihemolytic activity of anthocyanins is higher in O RhD−ve blood type (88.65 ± 2.23%) compared to O RhD+ve blood type (82.52 ± 2.87%). This suggests that anthocyanins could have a more pronounced protective effect against free radical-induced hemolysis in individuals with O RhD−ve blood type. The values of the photoprotective activity of anthocyanins on the action of photohemolysis induced by both ultraviolet radiation (UV-A and UV-B) are higher in blood type O RhD+ve (63.45 ± 3.12 and 60.34 ± 2.84%, respectively) compared to blood type O RhD−ve (40.15 ± 3.62 and 38.45 ± 2.64%, respectively). This suggests that anthocyanins may offer enhanced protection against ultraviolet radiation-induced damage in individuals with O RhD+ve blood type. Notably, the photoprotective activity against UV-A is substantially higher compared to UV-B for both blood types, suggesting that anthocyanins may offer better protection against longer wavelength ultraviolet radiation (UVA) than shorter wavelength ultraviolet radiation (UV-B).

After evaluating antihemolytic and photoprotective activities of anthocyanins, it is essential to highlight their differences. Antihemolytic activity prevents erythrocyte lysis by chemically induced radicals, while photoprotection mitigates UV-induced damage. Their effectiveness varies by blood type: stronger antihemolysis in O RhD− and greater UVA protection in O RhD+. These complementary effects expand understanding of anthocyanins’ role as protective agents against oxidative stressors [29,30].

Ultraviolet radiation, particularly UVA (315–395 nm) and UVB (250–315 nm), induces oxidative stress by generating reactive oxygen species (ROS) such as singlet oxygen (^1^O_2_), superoxide (O_2_•^−^), hydroxyl radicals (•OH), and hydrogen peroxide (H_2_O_2_), which damage lipids, proteins, and nucleic acids. UVB causes direct DNA damage, including thymine dimers, triggering inflammation, apoptosis, and repair, potentially leading to premature aging and cancer. Anthocyanins exhibit stronger photoprotection against UVA, as they absorb longer wavelengths and reduce ROS formation. Once biomolecules absorb UV, electrons shift to higher energy states, disrupting bonds and initiating radical chain reactions that amplify oxidative stress and cellular injury [31,32,33].

Therefore, understanding how anthocyanins interact with different radiation types and blood phenotypes is essential to optimizing their use in protecting cells from oxidative damage, supporting their potential application in functional foods and preventive health strategies against environmental stressors [34,35]. The observed antioxidant and erythroprotective effects may be largely attributed to cyanidin derivatives, the predominant anthocyanins in açaí, whose flavylium cation structure confers a strong ability to donate hydrogen atoms or electrons, thereby neutralizing reactive oxygen species (ROS) such as superoxide and peroxyl radicals. The multiple hydroxyl groups at positions 3, 5, and 7, together with the ortho-dihydroxylation in the B-ring (catechol group), enhance radical scavenging capacity through resonance stabilization of the resulting semiquinone. In erythrocytes, these structural features help maintain membrane integrity by preventing lipid peroxidation and reducing hemoglobin oxidation. Furthermore, phenolic acids, flavonoids, tocopherols, and phytosterols act synergistically, reinforcing antioxidant protection and contributing to cellular stability under oxidative stress [9,10].

#### 2.1.3. Morphological Alterations of Erythrocytes Under Oxidative Stress

**Figure 1:**
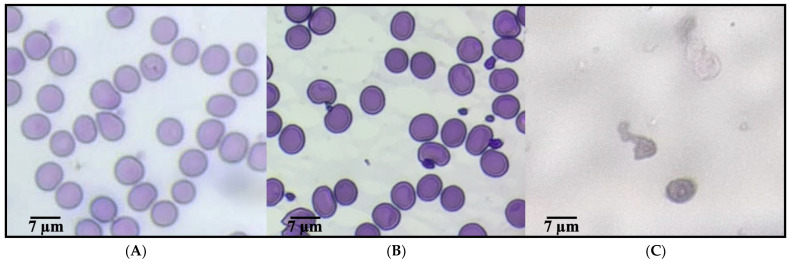
Evaluation of the erythroprotective potential of a yogurt formulation enriched with anthocyanin-loaded nanoliposomes in human erythrocytes exposed to oxidative stress. (**A**) Healthy erythrocytes (negative control). (**B**) Erythrocytes treated with a yogurt formulation enriched with anthocyanin-loaded nanoliposomes, indicating a protective effect against oxidative damage. (**C**) Erythrocytes exposed to AAPH, a free radical generator.

Figure 1 presents representative micrographs of human erythrocytes under different conditions, assessing the erythroprotective potential against oxidative stress induced by AAPH. Image (A), corresponding to the negative control, shows erythrocytes with characteristic biconcave morphology, well-preserved structure, and no signs of damage, indicative of a healthy physiological state in the absence of oxidative agents. In contrast, image (C) reveals the morphological alterations caused by AAPH exposure, including spherocytosis, cell fragmentation, and aggregation, all of which are typical features of severe oxidative damage. Notably, image (B) displays erythrocytes treated with the anthocyanin-nanoliposome, which retain a morphology closely resembling that of healthy cells, with minimal membrane damage and structural deformation. These findings suggest a pronounced protective effect, likely mediated by the antioxidant capacity of anthocyanin and its controlled release from nanoliposomes, supporting its potential use as a functional ingredient to counteract oxidative stress in erythrocytes [36].

Free radicals generated by AAPH (2,2′-azobis(2-amidinopropane) dihydrochloride), such as peroxyl radicals (ROO•) and alkoxyl radicals (RO•), induce oxidative stress by attacking the unsaturated components of the lipid bilayer of the plasma membrane, particularly the polyunsaturated fatty acids present in phospholipids. These radicals trigger redox (oxidation-reduction) reactions, initiating a lipid peroxidation chain: the ROO• radical abstracts a hydrogen atom from membrane lipids (LH), generating a new lipid radical (L•) and a lipid hydroperoxide (LOOH), which alters membrane fluidity and permeability, ultimately compromising structural integrity. Anthocyanins, as potent phenolic antioxidants, interrupt this oxidative chain through a hydrogen atom or electron donation mechanism. Chemically, the flavonoid structure of anthocyanins contains hydroxyl (-OH) groups on their aromatic rings, which donate a hydrogen atom (H•) to the ROO• radical, neutralizing it and forming a more stable species (ROOH), while the anthocyanin itself is converted into a relatively stable and resonance-stabilized radical that does not propagate the oxidative chain. This inhibition mechanism, mediated by Hydrogen Atom Transfer (HAT) or Single Electron Transfer (SET), plays a crucial role in preserving the structure and function of the cell membrane under oxidative stress conditions [30,37,38,39].

### 2.2. Evaluation of Encapsulation Performance and Controlled Release Behavior of Anthocyanin-Loaded Nanoliposomes

#### 2.2.1. Encapsulation Efficiency

The encapsulation efficiency (EE) of ANT-LN refers to the proportion of anthocyanins retained within the lipid vesicles relative to the total amount used during the encapsulation process. In other words, it measures how much of the bioactive compound—anthocyanins, in this case, was effectively incorporated into the nanoliposomes compared with the initial amount added. EE is a key parameter for assessing the potential application of nanoliposomes. In this study, the EE obtained was 91.34 ± 1.34%. Due to their polar nature, anthocyanins are primarily entrapped in the aqueous core of the vesicles, which can sometimes result in lower EE compared with lipophilic compounds. For example, hydrophobic molecules such as astaxanthin typically achieve encapsulation efficiencies around 97% [40,41,42]. Their affinity for the lipid bilayer allows them to be retained between the hydrophobic tails of the membrane, favoring more efficient entrapment. Nevertheless, the EE observed in this study is considered high and efficient, confirming that a substantial proportion of anthocyanins was successfully encapsulated. Such high EE is desirable to ensure effective delivery and controlled release in both drug delivery systems and functional food applications [42].

#### 2.2.2. Assessment of Particle Size and Zeta Potential Stability

The characterization of anthocyanin-loaded nanoliposomes presented in Table 1 demonstrates that the system possesses optimal physicochemical attributes for stability and bioactivity. The high encapsulation efficiency (91.34 ± 1.34%) reflects the strong affinity between anthocyanin molecules and the phospholipid bilayer, where hydrophobic aromatic rings and partially hydrophilic hydroxyl groups interact with both the lipid tails and polar head groups, favoring their entrapment. The small mean particle size (132 ± 14.23 nm) not only enhances colloidal stability but also facilitates cellular uptake, as vesicles below 200 nm are more likely to cross biological membranes via endocytosis. The low polydispersity index (0.21 ± 0.03) confirms a narrow size distribution, indicating that the preparation method successfully generated a homogeneous population of vesicles with reduced aggregation tendency. This homogeneity is critical for reproducible bioactivity and controlled release. Furthermore, the zeta potential value (−34.6 ± 1.8 mV) places the system within the range considered electrostatically stable (≥|30| mV), ensuring repulsion between vesicles and preventing flocculation.

Compared to previous studies, such as those by Guldiken et al. [23], Jiao et al. [43], and Chi et al. [44] which reported encapsulation efficiencies between 70 and 85% for anthocyanins with particle sizes above 200 nm, the present formulation exhibits superior performance, highlighting the quality of the lipid matrix and its ability to accommodate polyphenolic structures. Interestingly, Jiao et al. [43] demonstrated that surface modification of lutein-loaded nanoliposomes with polypeptides (PLL) reduced zeta potential values from −38.6 mV in undecorated systems to −27.9 mV, while significantly enhancing encapsulation efficiency. This finding reinforces the relevance of charge modulation and surface engineering as strategies to optimize stability and loading capacity, in line with the improved performance observed in the present formulation. At the molecular level, stabilization arises from hydrogen bonding between anthocyanin hydroxyl groups and phospholipid phosphate moieties, as well as hydrophobic interactions anchoring the anthocyanin aglycone into the bilayer core. This dual interaction pattern enhances retention, reduces premature degradation, and supports sustained release. Overall, the results indicate that these nanoliposomes are high-quality carriers with strong potential for nutraceutical and biomedical applications.

**Figure 2:**
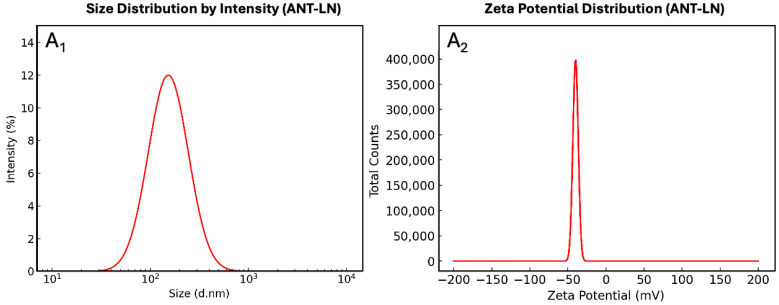
Particle size distribution by intensity (**A1**) and zeta potential distribution (**A2**) of anthocyanin-loaded nanoliposomes.

Figure 2A1,A2 depict the physicochemical characterization of anthocyanin-loaded nanoliposomes, revealing properties that highlight the quality and stability of the system. In Figure 2A1, the particle size distribution is centered around 153 nm, with a narrow and well-defined curve, reflecting the low polydispersity of the system. This homogeneity is a key indicator of efficient preparation, as the absence of multiple peaks suggests that the vesicles are morphologically uniform, with no significant secondary populations or aggregates. From a physicochemical perspective, this behavior can be explained by the favorable interaction of anthocyanins with the lipid bilayer, where aromatic hydroxyl groups establish hydrogen bonds with the polar head groups of phospholipids, while the more hydrophobic regions orient toward the membrane interior, promoting stable integration.

Conversely, Figure 2A2 shows a zeta potential of −34.6 mV, a sufficiently high magnitude to ensure colloidal stability through electrostatic repulsion between vesicles. This negative surface charge can be attributed both to the phosphate groups of the lipids and to the interactions with ionized phenolic groups of anthocyanins under physiological pH, thereby preventing liposome coagulation or fusion during storage. Taken together, these results demonstrate that the nanoformulation achieves high encapsulation efficiency, strong colloidal stability, and homogeneous distribution, features that enhance compound protection against degradation and support its potential applicability in functional foods or nutraceutical formulations.

#### 2.2.3. Aqueous Dispersibility Assay

The aqueous dispersibility of anthocyanin-loaded nanoliposomes (ANT-LN) was evaluated to assess their stability in liquid systems. The assay showed that anthocyanins encapsulated within nanoliposomes reached an apparent concentration of 2.7 µg/mL, normalized with respect to the anthocyanin content determined in the encapsulation efficiency assay. This finding indicates that ANT-LN are readily dispersible in water, which is a critical parameter for their application in food formulations. High dispersibility facilitates uniform distribution of the bioactive compound in aqueous matrices such as yogurt, beverages, or smoothies, thereby supporting consistent delivery of anthocyanin-associated benefits. In addition, dispersibility is closely related to bioaccessibility and bioavailability, as it promotes effective release and absorption during gastrointestinal digestion [1]. It should be noted that dispersibility can be influenced by lipid composition, particle size, surface charge, and storage conditions. Therefore, monitoring this parameter during formulation and storage is essential to guarantee the stability and functionality of ANT-LN. Overall, these results highlight the potential of anthocyanin-loaded nanoliposomes as effective delivery systems for incorporation into dairy-based functional foods [4].

#### 2.2.4. In Vitro Release

**Figure 3:**
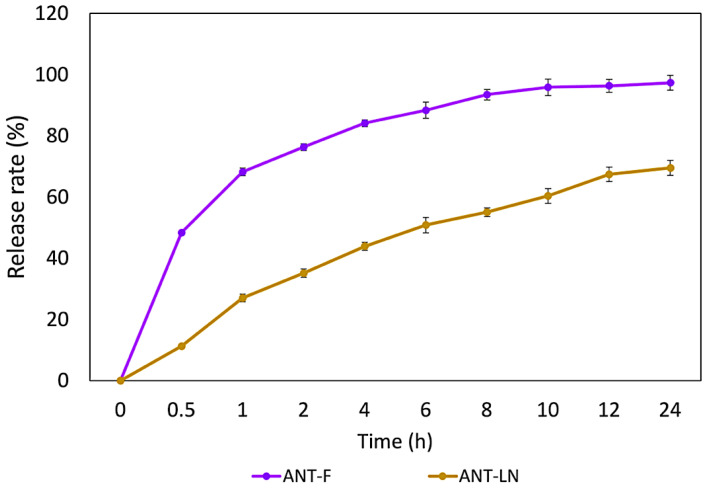
In vitro release of free anthocyanin solution (ANT-F) and anthocyanin-loaded nanoliposomes (ANT-LN) in PBS (pH 7.4) at 37 °C. Bars represent the mean ± standard deviation (n = 3).

The in vitro release profile of anthocyanin encapsulated in nanoliposomes and free anthocyanin solution represents a crucial index of the potential applications of nanoliposomes (Figure 3). The results of in vitro release of unencapsulated anthocyanin and anthocyanin-loaded nanoliposomes revealed significant differences in the amount of released anthocyanin. While unencapsulated anthocyanin showed a release of 93.34%, anthocyanin-loaded nanoliposomes exhibited a release of 64.32%. These findings suggest that encapsulation in nanoliposomes reduces the release of anthocyanin compared to the unencapsulated form [40]. The profile of anthocyanin release from nanoliposomes shows a clear difference compared to the release of anthocyanin from a pure solution. The release of anthocyanin from nanoliposomes was significantly slower at all evaluated time points compared to the pure anthocyanin solution. After 8 h, the release rate of anthocyanin from nanoliposomes was 43%, whereas the release rate of the pure anthocyanin solution was much higher, reaching 80%. These findings suggest that nanoliposomes act as controlled release vehicles for anthocyanin, meaning that the encapsulated anthocyanin is released gradually compared to the pure anthocyanin solution.

Previous studies on astaxanthin have shown a low release rate from nanoliposomes after 24 h (28.74%), compared to 64.32% for anthocyanins in our study, confirming the ability of nanoliposomes to provide sustained release and maintain stable plasma levels of bioactive compounds [4,5,40]. These differences are explained by the polarity of the molecules: astaxanthin, being lipophilic, integrates into the lipid bilayer and is slowly released, while anthocyanins, more hydrophilic, are encapsulated in the aqueous core and interact with both phases, resulting in a faster but still prolonged release. This structural behavior affects release kinetics and highlights the versatility of nanoliposomes for encapsulating molecules with different polarities. Furthermore, nanoliposomes not only regulate release but also protect anthocyanins from oxidative degradation, reinforcing their potential in food and drug delivery systems [44,45,46,47,48,49]. Similar sustained release profiles have been reported by Robles-García et al. [19] for fucoxanthin, Pan et al. [40] for astaxanthin, and Jiao et al. [50] for lutein, where encapsulation significantly reduced the release rate compared to free compounds. Collectively, these results confirm nanoliposomes as protective vehicles that extend bioactive stability and functionality. Thus, anthocyanin-loaded nanoliposomes (ANT-LN) emerge as a promising strategy for functional foods with enhanced nutraceutical properties.

#### 2.2.5. Controlled Release Kinetics Korsmeyer–Peppas

**Figure 4:**
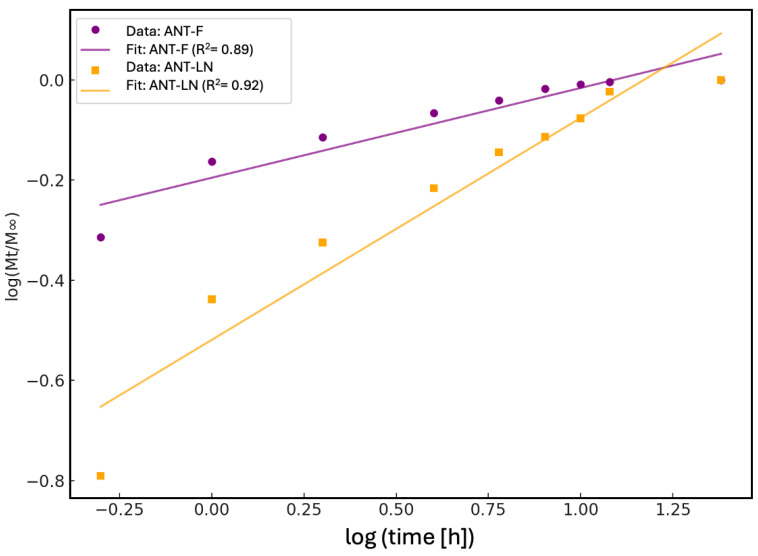
Fitting of anthocyanin release profiles to the Korsmeyer–Peppas kinetic model. The lines represent the corresponding linear fits, with determination coefficients (R^2^) of 0.89 for free anthocyanin solution (ANT-F) and 0.92 for anthocyanin-loaded nanoliposomes (ANT-LN), indicating a good fit to the model and highlighting the controlled release behavior provided by the nanoliposomes.

The kinetic analysis of the anthocyanin release profile using the Korsmeyer–Peppas model enabled the evaluation of the release mechanism of phenolic compounds in both their free form (ANT-F) and when encapsulated in nanoliposomes (ANT-LN) (Figure 4). This model, commonly applied to controlled release systems, is based on the relationship between the fraction of compound released (Mt/M∞) and time, represented on a logarithmic scale. The fitted kinetic parameters indicated that the ANT-F formulation exhibited a release exponent of n = 0.179, suggesting a mechanism consistent with Fickian diffusion, typical of systems where the compound is rapidly released without structural constraints. In contrast, the ANT-LN formulation showed a value of n = 0.443, indicating an anomalous or non-Fickian release process, where diffusion is influenced by the structure of the liposomal carrier and its interaction with the release medium. This difference underscores the role of nanoliposomes as controlled release systems capable of modulating water access to the encapsulated compound and extending its release over time.

The determination coefficients (R^2^) obtained for both models strongly support the suitability of the Korsmeyer–Peppas model for the experimental data. For the free anthocyanin (ANT-F), the R^2^ was 0.89, indicating a good fit, albeit with slight deviations associated with immediate and unrestricted release. On the other hand, the nanoencapsulated anthocyanin (ANT-LN) showed an R^2^ of 0.92, reflecting an excellent fit to the model and confirming the sustained and controlled release behavior of the compounds when protected by the liposomal matrix. These results not only demonstrate the ability of nanoliposomes to function as effective controlled release systems but also enable the prediction of their performance in food or pharmaceutical applications where stability, biological activity, and bioavailability of phenolic compounds such as anthocyanins are critical. Overall, the application of the Korsmeyer–Peppas model serves as a fundamental tool to describe, interpret, and optimize nanoengineered sustained-release systems [1,51].

#### 2.2.6. Stability of ANT-LN by Centrifugation

The stability of anthocyanin-loaded nanoliposomes, as measured by centrifugation, exhibited a high retention percentage (92.67 ± 1.25%). This outcome indicates that the nanoliposomes maintained their structural integrity and retained most of the encapsulated anthocyanins after being subjected to centrifugal forces. Centrifugation is a technique commonly employed to assess the stability of nanoencapsulation systems as it simulates mechanical stress conditions that could occur during the processing, storage, or handling of food products. In this case, the high stability percentage observed suggests that the nanoliposomes are robust and capable of withstanding these adverse conditions [52].

These results regarding the retention of encapsulated anthocyanins are promising, indicating that the majority of bioactive compounds remained within the nanoliposomes and did not degrade during the centrifugation process. This implies that nanoliposomes effectively protect and retain anthocyanins, which is crucial to ensure their efficacy as delivery vehicles in food applications. The findings support the suitability of nanoliposomes as encapsulation systems for anthocyanins and suggest they could be an effective strategy to enhance the stability and bioavailability of these compounds in food products. However, it is important to note that stability measured by centrifugation is only one aspect of assessing the long-term stability of nanoliposomes in real food applications; thus, further studies would be needed to confirm their viability under practical conditions [53].

### 2.3. Morphological Characterization of Anthocyanin-Loaded Nanoliposomes in Enriched Yogurt

**Figure 5:**
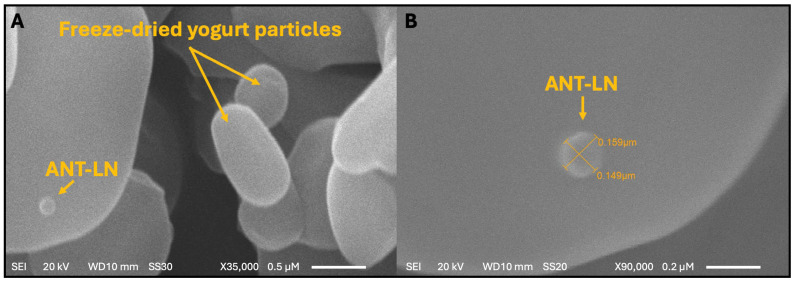
Morphology and particle size of anthocyanin-loaded nanoliposomes (ANT-LN) in lyophilized yogurt observed by scanning electron microscopy (SEM). (**A**) Magnification: ×35,000; scale bar = 0.5 µm. (**B**) Magnification: ×90,000; scale bar = 0.2 µm.

The morphological characterization of anthocyanin-loaded nanoliposomes (Ant-LN) was carried out using scanning electron microscopy (SEM) to assess their structural integrity, uniformity, and successful incorporation into the yogurt matrix (Figure 5). SEM analysis revealed well-defined, spherical lipid vesicles with a homogeneous size distribution, both in their unloaded and anthocyanin-loaded forms. These liposomal vesicles exhibited no signs of deformation, aggregation, or leakage—features that are indicative of ideal nanoliposomes. Their spherical morphology contributes to enhanced structural stability, while the absence of clustering ensures uniform dispersion, essential for their functionality as delivery vehicles [40,54].

The improved stability and bioavailability of these systems are particularly relevant when integrated into complex food matrices like yogurt. Upon incorporation, ANT-LN nanoliposomes were found to be stably embedded within the yogurt matrix, maintaining their structural integrity throughout. The vesicles maintained a consistent size, ranging from 0.149 to 0.159 µm, reflecting excellent preservation of their morphology and structural integrity even in the complex dairy matrix. This uniform distribution is critical for ensuring homogeneity of the bioactive compounds and for safeguarding the anthocyanins during storage and digestion. The demonstrated stability of ANT-LN in the yogurt matrix underscores their robustness against food processing and storage conditions, supporting their application in functional food development. These findings collectively validate the feasibility of anthocyanin-loaded nanoliposomes as effective delivery systems in enriched yogurt, offering therapeutic potential—particularly for brain health—while enhancing consumer health benefits [40,55,56].

### 2.4. Proximate Composition of Yogurt Enriched with Anthocyanin-Loaded Nanoliposomes

The proximate composition of yogurt enriched with anthocyanin-loaded nanoliposomes exhibited notable variations compared to the control yogurt, particularly in dry matter, protein, fat, and carbohydrate content (Table 2). The most significant increase in dry matter was observed in the yogurt with 5% nanoliposomes (Y-ANT-5), reaching 15.8%, compared to 11.6% in the control (Y-C). This could be attributed to the incorporation of the lipid phase and solid content derived from the nanoliposome formulation. This increase was accompanied by a relative decrease in moisture (84.2% vs. 88.4%). The slight elevation in protein content in Y-ANT-5 (37.2%) may be explained by the presence of protein-based encapsulating agents (such as casein or plant protein isolates), which become integrated into the yogurt matrix. Likewise, the fat content increased slightly in both nanoliposome-enriched treatments, likely due to the structural lipids used in vesicle formulation (e.g., phospholipids), suggesting that encapsulation not only acts as a vehicle for bioactive delivery but also modifies the food’s bromatological profile [57].

On the other hand, the carbohydrate content decreased markedly in Y-ANT-5 (21.9%) compared to the control (26.0%), possibly due to the displacement of non-encapsulated solids by the lipid and protein components of the nanoliposomes, thereby altering the relative nutrient proportions. Interestingly, in the yogurt with 10% nanoliposomes (Y-ANT-10), the values for dry matter, moisture, and protein tend to revert toward those of the control, which may indicate that at higher concentrations, a saturation effect or physicochemical instability may occur, reducing the integration efficiency of the nanoliposomal system into the yogurt matrix. Additionally, ash content showed a slight increase in the enriched treatments, potentially due to mineral contributions from the dispersion medium or the encapsulated compounds. The incorporation of nanoliposomes positively impacts the stability of the food matrix by enhancing the retention of bioactive compounds, reducing syneresis, and reinforcing the yogurt’s protein network. However, elevated concentrations may induce physicochemical instability, negatively affecting the product’s texture and homogeneity during storage [57].

When comparing the proximate composition of yogurt enriched with anthocyanin- versus fucoxanthin-loaded nanoliposomes [19], key differences arise. Both systems increased dry matter and fat while reducing carbohydrates and moisture. However, anthocyanin-loaded nanoliposomes significantly raised protein content at 5%, suggesting higher protein encapsulant integration, unlike fucoxanthin formulations, which maintained stable protein levels and showed more consistent trends across concentrations. Ash content increased in both, likely due to the presence of minerals in the nanocarriers. These findings highlight how the type of bioactive and encapsulation matrix differentially modulate the nutritional and technological properties of fortified yogurts.

### 2.5. Microbiological Assessment of Artisanal Yogurt

The microbiological quality assessment of yogurt formulations enriched with anthocyanin-loaded nanoliposomes revealed that all samples remained within the microbiological safety standards stipulated by NOM-243-SSA1-2010 for fermented milk products [58]. The enumeration of mesophilic aerobic microorganisms indicated viable microbial populations characteristic of proper fermentation processes (1.0 × 10^6^ CFU/g) [59]. Specifically, the control yogurt (Y-C) registered a microbial count of 1.4 × 10^7^ CFU/g, while the yogurts containing 5% (Y-ANT-5) and 10% (Y-ANT-10) nanoliposomes exhibited slightly lower values of 9.8 × 10^6^ and 8.4 × 10^6^ CFU/g, respectively. These results suggest that, although nanoliposome enrichment caused a modest reduction in microbial load, all values remained above the minimum threshold (1.0 × 10^6^ CFU/g), ensuring an active population of lactic acid bacteria essential for fermentation, sensory development, and potential probiotic functionality [60].

The slight decline in mesophilic counts with higher nanoliposome concentration could be attributed to the mild antimicrobial or antioxidant properties of the anthocyanins or carrier system, which may exert subtle effects on bacterial proliferation without compromising the viability of beneficial microbes [61]. Additionally, the absence of total and fecal coliforms in all formulations confirms the hygienic production practices and appropriate fermentation control during yogurt elaboration. No fungal contamination was detected, as evidenced by the absence of molds and yeasts, underscoring the effectiveness of both the cold storage conditions and the protective microenvironment provided by the nanoliposomes [61,62]. Overall, the data demonstrate that anthocyanin-loaded nanoliposome incorporation does not adversely affect the microbiological integrity of the yogurt matrix and supports its safe consumption and functional quality during refrigerated storage [5,40,55].

### 2.6. Effect of Cold Storage Conditions on the Physicochemical and Rheological Properties of Yogurt Enriched with ANT-LN

#### 2.6.1. pH

**Figure 6:**
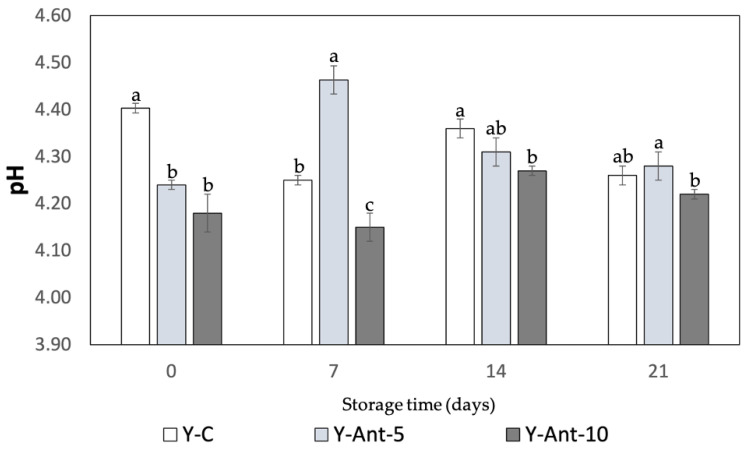
Effect of anthocyanin-loaded nanoliposome addition and cold storage time on the pH of yogurt formulations. All data were analyzed using two-way ANOVA with interactions. Values followed by different lowercase letters (a–c: pH effects) within the same treatment indicate statistically significant differences (*p* < 0.05). Y-C = Control Yogurt without nanoliposomes; Y-Ant-5 = Yogurt enriched with 5% nanoliposomes; Y-Ant-10 = Yogurt enriched with 10% nanoliposomes. Bars represent the standard deviation of at least three replicates (n > 3) per concentration.

The pH of yogurt is a key indicator of its quality, stability, and safety (Figure 6). In this study, all formulations, control (Y-C) and those enriched with 5% and 10% anthocyanin-loaded nanoliposomes (Y-ANT-5 and Y-ANT-10), showed minor variations in pH during refrigerated storage. Initially, the control yogurt exhibited a slightly higher pH than the enriched variants, suggesting a mild acidifying effect from the nanoliposomes, though this difference was not practically significant. Throughout the 21-day storage period, all samples-maintained pH values within a narrow and acceptable range. Y-C showed a slight decrease from 4.40 to 4.26, while Y-ANT-5 and Y-ANT-10 experienced modest fluctuations, with temporary increases during the first week followed by gradual declines, ending at 4.31 and 4.22, respectively. These patterns reflect typical fermentation dynamics and indicate that the incorporation of nanoliposomes did not compromise the pH stability of the yogurt [63].

The addition of anthocyanin-loaded nanoliposomes did not significantly affect yogurt acidity during storage, as all formulations-maintained pH values within the typical 4.0–4.6 range. This suggests microbiological safety and acceptable sensory quality. However, further studies are recommended to evaluate potential long-term impacts on yogurt stability and overall product quality [64,65,66]. The studies by Robles-García et al. [19] and Anuyahong et al. [14] reveal differing impacts of bioactive compounds on yogurt pH. Fucoxanthin-loaded nanoliposomes altered pH dynamics during storage, with 5% accelerating acidification and 10% showing stabilization, likely due to buffering effects. In contrast, blueberry rice extracts delayed acidification during fermentation but did not affect final pH. Yogurts enriched with anthocyanin-loaded nanoliposomes showed minimal pH fluctuations, indicating that nanoliposomes act as inert carriers that preserve acidity during storage. These findings suggest that the delivery system plays a key role in modulating yogurt acidification beyond the bioactive compound itself [67].

#### 2.6.2. Titratable Acidity

**Figure 7:**
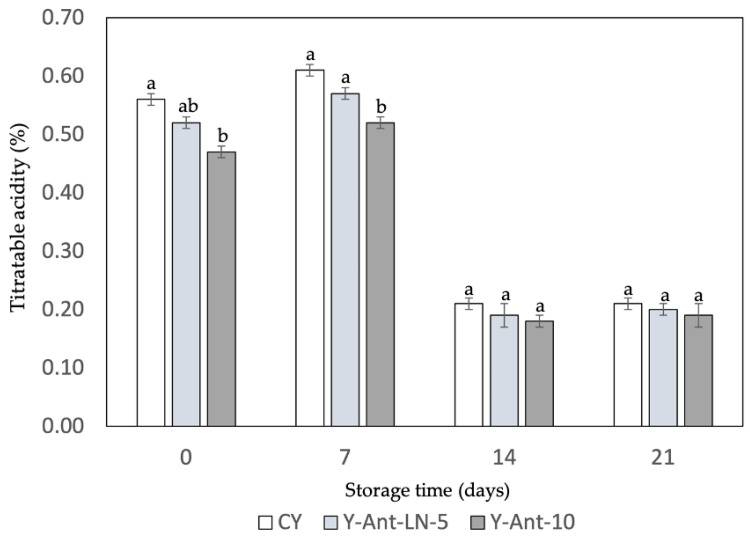
Effect of anthocyanin-loaded nanoliposome addition and cold storage time on the titratable acidity of yogurt formulations. All data were analyzed using one-way ANOVA with interactions. Values followed by different lowercase letters within the same treatment indicate statistically significant differences (*p* < 0.05). Y-C = Control Yogurt without nanoliposomes; Y-Ant-5 = Yogurt enriched with 5% nanoliposomes; Y-Ant-10 = Yogurt enriched with 10% nanoliposomes. Bars represent the standard deviation of at least three replicates (n > 3) per concentration.

Titratable acidity is a key indicator of yogurt quality and stability. Figure 7 shows a gradual decrease in acidity across all formulations during storage. Initially, the control yogurt (Y-C) had the highest titratable acidity (0.56%), followed by samples enriched with 5% and 10% anthocyanin-loaded nanoliposomes (Y-ANT-LN-5% and Y-ANT-LN-10%), suggesting that anthocyanin incorporation may slightly reduce initial acidity. As storage progresses, all formulations exhibit a decline in acidity; however, enriched yogurts tend to maintain slightly higher levels than the control, possibly due to the stabilizing influence of anthocyanins on lactic acid bacteria activity. By day 14, titratable acidity decreased notably in all samples, but enriched yogurts still showed marginally lower reductions, indicating potential stabilization. On day 21, acidity values among all samples were more uniform, with enriched yogurts retaining slightly higher values than the control. These findings suggest that anthocyanins may confer protective effects on yogurt acidity during storage, enhancing product stability, though further research is required to elucidate the underlying mechanisms.

The data suggest that the addition of anthocyanin-loaded nanoliposomes may have a minimal effect on the titratable acidity of yogurt during the storage period. However, further research is needed to fully understand how these formulations affect the quality and stability of yogurt over time. Additionally, comparing these results with those of Robles-García et al. [19] and Anuyahong et al. [14] highlights the influence of different bioactive additives on titratable acidity in yogurt. Robles-García et al. [19] reported that fucoxanthin-loaded nanoliposomes slightly reduced initial acidity and stabilized it during 21 days of storage, possibly by modulating acid production through interactions with lactic acid bacteria. This behavior contributed to enhanced structural integrity and shelf life, with a milder acidity profile that may be more acceptable to certain consumers. In contrast, Anuyahong et al. [14] found that the addition of blueberry rice extract affected acidification during fermentation but not the final titratable acidity, suggesting limited long-term impact. These differences emphasize that the functional and technological effects of additives depend not only on the compound used but also on its delivery system [67]. Therefore, careful selection of bioactives and encapsulation methods is essential when formulating functional yogurts to ensure desirable acidity, stability, and consumer acceptability throughout storage [68,69].

#### 2.6.3. Syneresis Susceptibility

**Figure 8:**
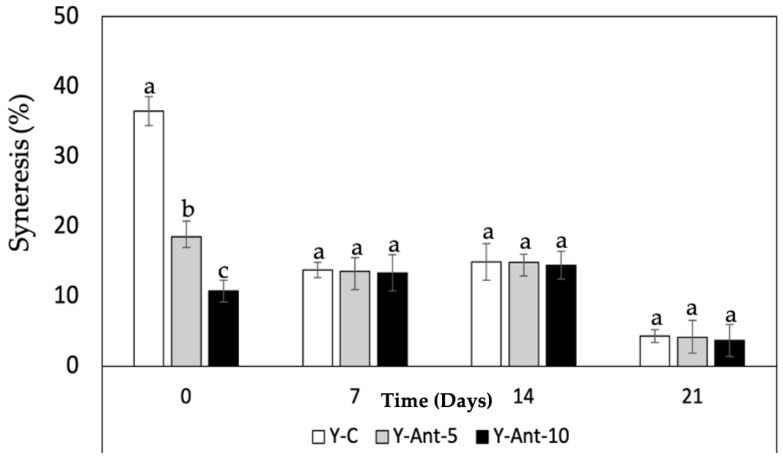
Effect of anthocyanin-loaded nanoliposome addition and cold storage time on the syneresis (%) of yogurt formulations. All data were analyzed using one-way ANOVA with interactions. Values followed by different lowercase letters (a–c: storage time effects) within the same treatment indicate statistically significant differences (*p* < 0.05). Y-C = Control Yogurt without nanoliposomes; Y-Ant-5 = Yogurt enriched with 5% nanoliposomes; Y-Ant-10 = Yogurt enriched with 10% nanoliposomes. Bars represent the standard deviation of at least three replicates (n > 3) per concentration.

For the samples on day 0, yogurt without nanoliposomes (Y-C) showed the highest syneresis, followed by Y-ANT-5% and Y-ANT-10%, indicating that nanoliposome addition reduces water separation (Figure 8). Over 21 days of storage, all formulations showed decreased syneresis, with the most significant reduction observed in Y-ANT-10%. By day 21, Y-C still exhibited the highest syneresis, while Y-ANT-10% had the lowest. These results suggest that incorporating 10% nanoliposomes significantly enhances yogurt stability by minimizing syneresis over time. Thus, Y-ANT-10% offers the best performance in preserving texture and water-holding capacity during refrigerated storage, supporting its use for improving the physicochemical quality of functional yogurts [6,9].

Syneresis refers to the separation of whey from the yogurt gel and reflects the structural balance within the casein network, which depends on its ability to maintain attractive forces and rearrange molecular bonds [70,71]. High levels of syneresis indicate poor structural integrity and reduced product quality. In studies by Robles-García et al. [19], the incorporation of fucoxanthin-loaded nanoliposomes significantly reduced syneresis over 21 days of cold storage. This improvement is attributed to interactions between nanoliposomes and casein micelles, reinforcing the gel matrix and enhancing water retention. As a result, the yogurt maintained a firmer texture, higher viscosity, and improved structural stability. These findings are consistent with previous studies showing that the addition of bioactive compounds such as phenols, flavonoids, and anthocyanins to yogurt can reduce syneresis [14]. This effect is likely related to polyphenols’ strong affinity for casein, forming soluble complexes that stabilize the gel matrix, limit whey release, and improve texture [67].

#### 2.6.4. Water-Holding Capacity (WHC)

**Figure 9:**
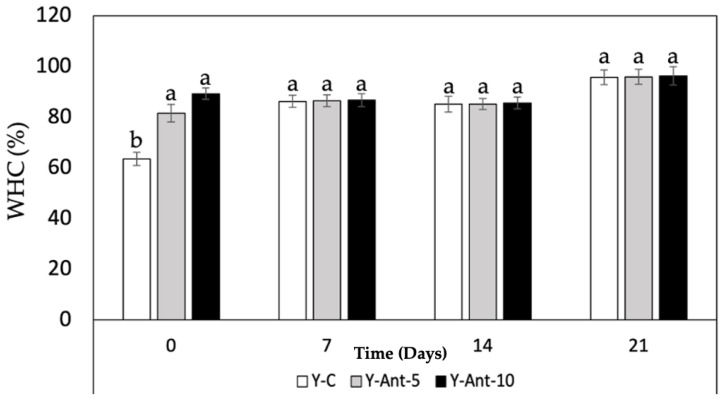
Effect of anthocyanin-loaded nanoliposome addition and cold storage time on the water-holding capacity (%) of yogurt formulations. All data were analyzed using one-way ANOVA with interactions. Values followed by different lowercase letters (a–b: storage time effects) within the same treatment indicate statistically significant differences (*p* < 0.05). Y-C = Control Yogurt without nanoliposomes; Y-Ant-5 = Yogurt enriched with 5% nanoliposomes; Y-Ant-10 = Yogurt enriched with 10% nanoliposomes. Bars represent the standard deviation of at least three replicates (n > 3) per concentration.

Based on the data provided in Figure 9, it can be observed that the water-holding capacity (WHC) increases over time for all formulations of yogurt enriched with anthocyanins (Y-ANT-5 and Y-ANT-10) and the control yogurt (Y-C). Overall, throughout the 21 days of storage, all formulations exhibit a progressive increase in WHC, indicating greater water retention within the yogurt matrix. Regarding the comparison between formulations, it is evident that both Y-ANT-5 (81–95%) and Y-ANT-10 (89–96%) demonstrate higher WHC compared to Y-C (63–95%) on all storage days. This suggests that the addition of anthocyanin-loaded nanoliposomes enhances the water-holding capacity of yogurt [14]. Furthermore, Y-ANT-10 exhibits the highest WHC compared to Y-ANT-5 on all storage days, indicating that a higher concentration of nanoliposomes might be associated with greater water retention capacity in yogurt.

In terms of quality associated with syneresis, it can be inferred that greater water-holding capacity is related to lower syneresis, as higher water retention within the yogurt matrix implies less serum release. Therefore, based on the WHC results, it could be suggested that both Y-ANT-5 and Y-ANT-10 have better quality associated with syneresis compared to the control yogurt (Y-C), with Y-ANT-10 showing the best quality in terms of water retention and potential reduction in syneresis. Thus, water-holding capacity (WHC) and susceptibility to syneresis are closely related in yogurts enriched with anthocyanin-loaded nanoliposomes [14,48]. Yogurt formulations with high WHC tend to experience reduced syneresis as they retain more water and consequently release less serum. Therefore, the yogurts with high water-holding capacity, such as those enriched with anthocyanin-loaded nanoliposomes, may have better quality in terms of texture and consistency due to reduced syneresis [72,73].

#### 2.6.5. Viscosity

**Figure 10:**
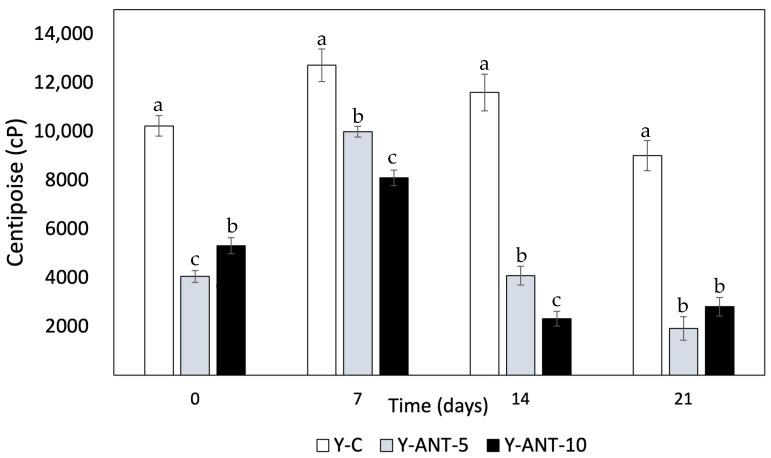
Effect of anthocyanin-loaded nanoliposome addition and cold storage time on the viscosity (cP) of yogurt formulations. All data were analyzed using one-way ANOVA with interactions. Values followed by different lowercase letters (a–c: storage time effects) within the same treatment indicate statistically significant differences (*p* < 0.05). Y-C = Control Yogurt without nanoliposomes; Y-Ant-5 = Yogurt enriched with 5% nanoliposomes; Y-Ant-10 = Yogurt enriched with 10% nanoliposomes. Bars represent the standard deviation of at least three replicates (n > 3) per concentration.

Viscosity data provide crucial information about the texture and consistency of yogurt, which can influence sensory perception and product quality [74]. Observing the results of Figure 10, it can be noted that on day 0, yogurt without nanoliposomes (Y-C) has the highest viscosity compared to formulations enriched with 5% and 10% nanoliposomes. This suggests that the addition of nanoliposomes may decrease the initial viscosity of yogurt. However, variations in viscosity are observed over storage time. Overall, a decrease in viscosity is observed for all formulations over time, which could be attributed to changes in the structure and composition of yogurt during storage. The decrease in viscosity in yogurt added with nanoliposomes may have different implications depending on the type of product and consumer preferences. The viscosity is associated with the texture and mouthfeel of dairy products. Therefore, the decrease in viscosity can have both positive and negative aspects on commercial quality and consumer perception, especially concerning set and drinkable yogurts [75].

Within the positive aspects, it can be noted that a decrease in viscosity may result in a smoother yogurt, making it easier to drink, especially for consumers preferring dairy products with a less dense consistency. Simultaneously, it enhances sensory perception. Some consumers may perceive a softer and lighter texture in yogurt with lower viscosity, thereby improving the consumption experience and product appeal [76]. Yogurts with lower viscosity may also be easier to dispense or pour, which could be preferred by consumers seeking convenience in their daily consumption. Conversely, some negative attributes of generating yogurt with improved viscosity can be highlighted. In some cases, consumers may associate lower viscosity with inferior quality, especially if they are accustomed to denser and creamier yogurts. For consumers preferring yogurts with a thicker and creamier texture, a decrease in viscosity may result in lower sensory satisfaction and a perception that the product is less indulgent [75,77].

In set yogurts, a significant decrease in viscosity may compromise shape and cohesion, affecting consumer perception and experience. While reduced viscosity in yogurt enriched with nanoliposomes may depend on product type and individual preferences, it remains crucial for manufacturers to evaluate these factors through market testing before launching new products [76]. Viscosity is also linked to yogurt’s ability to retain water; lower viscosity often suggests higher susceptibility to syneresis. In this context, yogurt without nanoliposomes (Y-C) tends to exhibit higher viscosity than enriched formulations, implying better serum retention. However, viscosity is not the only factor influencing syneresis—water-holding capacity, gel structure, and interactions with nanoliposomes also contribute significantly [9]. Previous studies by Anuyahong et al. [14], Liu and Lv [74], and Athar et al. [78] support that increased viscosity relates to higher total solids and gel firmness, reducing syneresis. Additionally, nanoemulsion production via optimized ultrasound processing can affect viscosity and final product characteristics. Overall, viscosity is a critical quality parameter in both yogurt formulation and nanoemulsion design.

#### 2.6.6. Texture (Firmness and Consistency)

**Figure 11:**
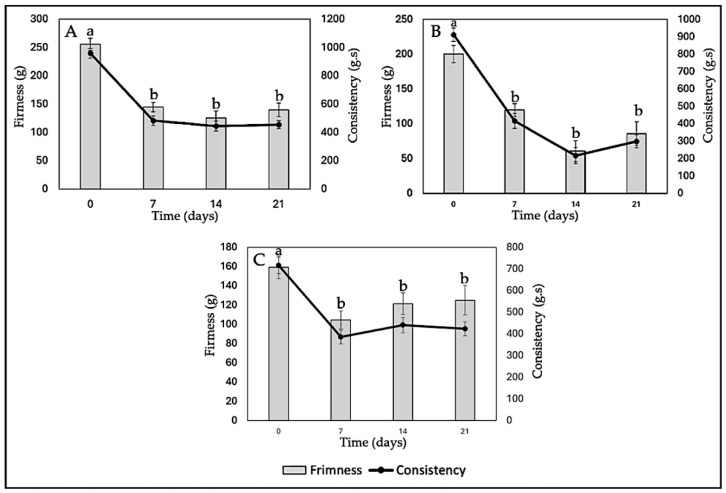
Effect of anthocyanin-loaded nanoliposome addition and cold storage time on the texture (Firmness and Consistency) of yogurt formulations. All data were analyzed using one-way ANOVA with interactions. Values followed by different lowercase letters (a–b: storage time effects) within the same treatment indicate statistically significant differences (*p* < 0.05). (**A**) Y-C: Control Yogurt; (**B**) Y-Ant-5; Yogurt-enriched with 5% of anthocyanin-loaded nanoliposome; (**C**) Yogurt-enriched with 10% of anthocyanin-loaded nanoliposome.

In Figure 11A, it is observed that both the firmness and consistency of yogurt without nanoliposomes (Y-C) decrease over storage time. This can be attributed to several factors, such as degradation of the yogurt gel structure during storage, serum release, and water loss. In terms of quality associated with the percentage of syneresis, lower syneresis is generally considered desirable in yogurt, as it indicates adequate water retention and a firmer gel structure. However, in this case, we do not have direct data on syneresis in the yogurt formulations enriched with anthocyanins (Y-ANT-5 and Y-ANT-10) to compare with yogurt without nanoliposomes (Y-C). To determine which formulation has better quality associated with its syneresis percentage, it would be necessary to directly analyze syneresis data for each formulation and compare them with firmness and consistency data. In the absence of such data, we cannot make a definitive assessment of which formulation is most favorable in terms of quality associated with syneresis [9,14,66].

The data from Figure 11B show that both the firmness and consistency of yogurt enriched with anthocyanins (Y-Ant-5) significantly decrease over the 21-day storage period. When comparing these values with those of yogurt without nanoliposomes (Y-C), it is evident that the firmness and consistency of yogurt enriched with anthocyanins are considerably lower at each time point. This suggests that the addition of anthocyanins, in this case, does not improve the firmness or consistency of yogurt compared to the non-enriched yogurt. Concerning syneresis, it can be expected that this formulation of yogurt enriched with anthocyanins may have a higher syneresis percentage due to the decrease in firmness and consistency [11,76].

Yogurt firmness refers to the force required to penetrate the yogurt structure with a probe. Generally, higher firmness is associated with greater density or cohesion of the yogurt structure [67]. It is observed that on day 0, Y-Ant-10 yogurt has a firmness of 159.28 g, while Y-C yogurt has a firmness of 255.59 g, indicating that yogurt without nanoliposomes is initially firmer (Figure 11C). However, over the 21-day period, the firmness of Y-Ant-10 yogurt experiences fluctuations, reaching a maximum value of 124.99 g on day 21. On the other hand, the firmness of Y-C yogurt gradually decreases over time, reaching a minimum value of 139.58 g on day 21. Yogurt consistency refers to its resistance to flow under the application of force [67]. On day 0, Y-Ant-10 yogurt has a consistency of 716.14 g·s, while Y-C yogurt has a consistency of 958.35 g·s. Over time, the consistency of Y-Ant-10 yogurt experiences a slight decrease, reaching a value of 423.19 g·s on day 21. On the other hand, the consistency of Y-C yogurt also decreases over time, reaching a value of 454.14 g·s on day 21.

**Figure 12:**
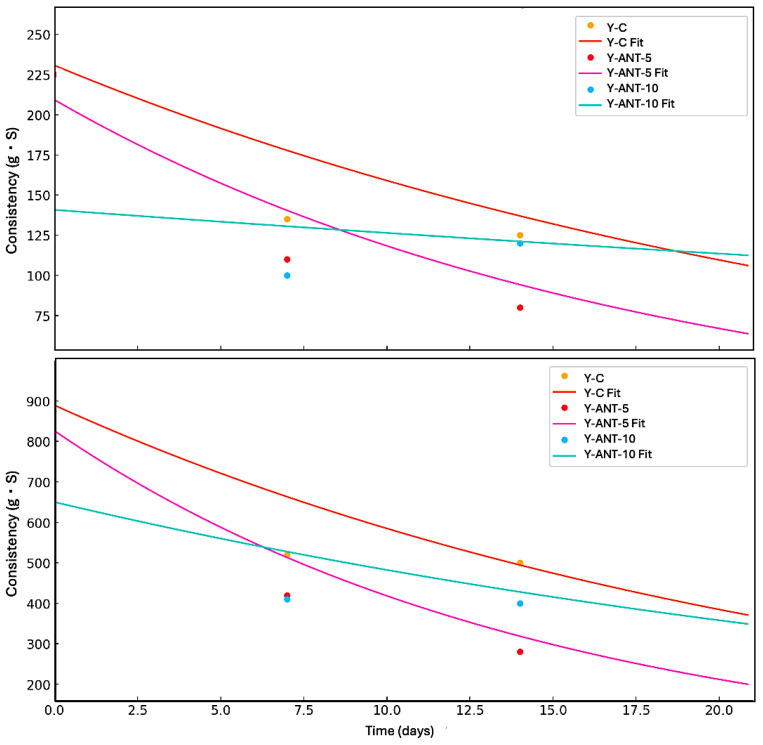
Kinetic modeling of the loss of firmness and consistency in yogurt formulations during refrigerated storage over 21 days. The curves represent first-order kinetic fits for control yogurt (Y-C), yogurt enriched with 5% anthocyanin-loaded nanoliposomes (Y-Ant-5), and yogurt enriched with 10% anthocyanin-loaded nanoliposomes (Y-Ant-10). Experimental data points are shown as markers, and fitted curves are represented by solid lines. The model describes an exponential decay behavior, indicating differences in structural degradation rates among formulations.

In Figure 12, the kinetic model of firmness and consistency loss in yogurt enriched with anthocyanin-loaded nanoliposomes (Y-Ant-5 and Y-Ant-10) and control yogurt (Y-C) is presented. The data were fitted to a first-order model, which describes an exponential decrease in these textural properties during storage. The experimental data estimated from the graphs showed that, although the control yogurt initially exhibited the highest firmness and consistency, it also experienced the fastest loss of these characteristics. In contrast, the Y-Ant-10 yogurt showed a slower decline in firmness and consistency, suggesting greater structural stability throughout storage. This behavior may be attributed to the stabilizing effect of nanoliposomes within the yogurt matrix. The application of the kinetic model enabled the estimation of degradation trends and allowed for the comparison of textural degradation rates between formulations. This approach is valuable for predicting shelf life and optimizing formulations with improved rheological performance in functional dairy products [67].

The inclusion of anthocyanin-loaded nanoliposomes contributes to improved structural stability, evidenced by the lower loss of texture properties during storage. This enhancement may positively impact consumer perception, as a creamy and stable texture is often preferred. Additionally, these nanoliposomes may provide antioxidant benefits and reduce susceptibility to syneresis. Thus, their incorporation not only supports textural preservation but could enhance the functional quality of yogurt. Nevertheless, further research is needed to fully elucidate the mechanisms involved and evaluate consumer acceptance and the product’s shelf life [11,67,77].

#### 2.6.7. Rheological Analysis

**Figure 13:**
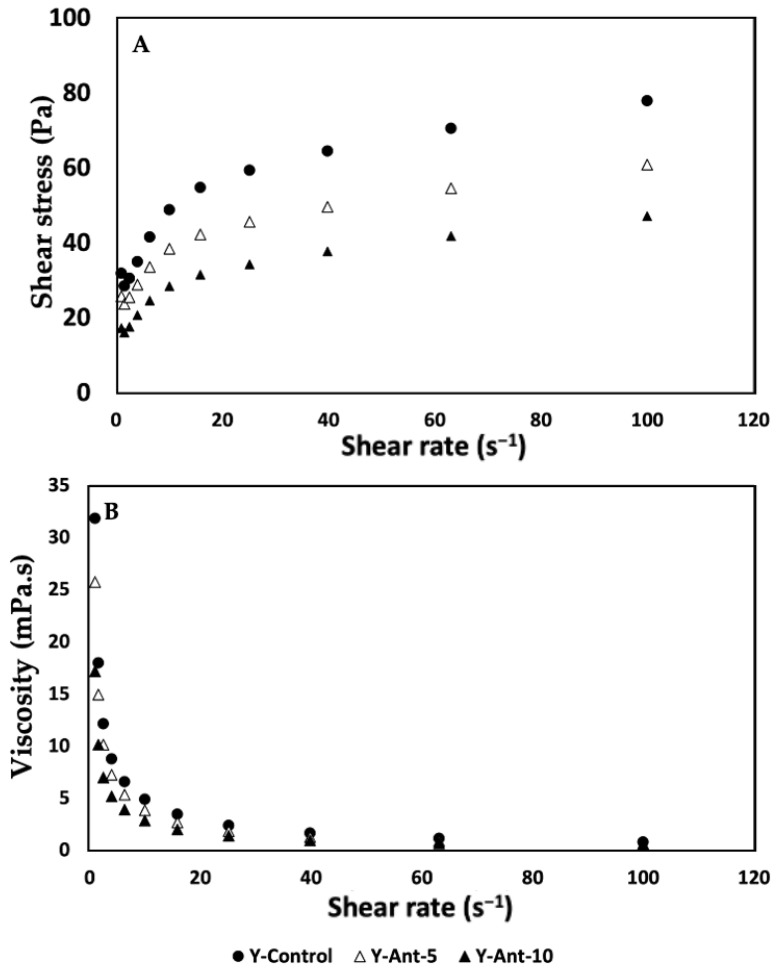
(**A**,**B**) Effect of the addition of nanoliposomes and cold storage time on the rheological properties (Shear resistance and viscosity) of yogurt formulations. All results were analyzed in triplicate (n > 3).

To analyze the results of shear resistance as a rheological property of anthocyanin-enriched yogurt formulations (Figure 13), we first observe that, as the concentration of anthocyanins increases, shear resistance decreases compared to the control. Specifically, formulation Y-Ant-10 exhibits the lowest shear resistance at all evaluated shear rate levels. This suggests that the addition of ANT-LN affects the shear resistance of yogurt. Considering the quality associated with shear response and rheological properties, the control yogurt, without enrichment, exhibits the best quality in terms of shear resistance. This is because it shows the highest resistance at all evaluated shear rate levels compared to the anthocyanin-enriched formulations [79]. Therefore, the control formulation would be considered the best option in terms of quality associated with shear response and rheological properties in this study [70].

Analyzing the shear resistance results (Figure 13A) together with the viscosity column, we observe an inverse relationship between shear resistance and viscosity in anthocyanin-enriched yogurt formulations. Formulations Y-Ant-5 and Y-Ant-10, which exhibit lower shear resistance compared to the control, also show lower viscosity. This suggests that anthocyanins have a negative effect on both shear resistance and yogurt viscosity. On the other hand, the control yogurt exhibits the highest shear resistance and, overall, the highest viscosity compared to the anthocyanin-enriched formulations. This indicates a direct relationship between shear resistance and viscosity, where yogurt with higher viscosity tends to have higher shear resistance. In terms of quality associated with shear response and rheological properties, the results suggest that the control yogurt, with its higher shear resistance and viscosity, would be the best option [6]. This implies that the control formulation would have a firmer and more consistent texture compared to the anthocyanin-enriched formulations. However, in technological terms, the consistency of enriched yogurt could lead to a drinkable yogurt formulation with greater opportunities in consumption and commercialization.

When analyzing the viscosity data (Figure 13B) of anthocyanin-enriched yogurt, a decrease in viscosity was observed as anthocyanin concentration increased, particularly in Y-Ant-10. Viscosity is closely related to texture and consistency; lower viscosity indicates a more fluid product, which may be preferred by consumers who enjoy drinkable yogurts. However, reduced viscosity also implies lower shear resistance and less firmness, potentially affecting yogurt quality. The control yogurt, with the highest viscosity, is therefore considered superior in terms of texture and consistency [80,81]. Ideal rheological properties depend on consumer preferences and product use. Yogurt should maintain a balance between firmness and softness, firm enough to hold shape, yet smooth and easy to consume. Adequate viscosity enhances creaminess and mouthfeel, contributing to a pleasant sensory experience [82]. Thus, viscosity plays a key role in consumer acceptance and overall quality of yogurt formulations.

Although the physicochemical and sensory parameters confirmed the quality of anthocyanin-loaded nanoliposome (ANT-LN) enriched yogurts, this study did not directly evaluate the stability of anthocyanins during cold storage. It has been reported that lactic acid bacteria (LAB) can metabolize anthocyanins in fermented milks, thereby reducing their concentration and antioxidant potential over time. This factor must be considered when interpreting our results, as the degradation of anthocyanins could have occurred despite their encapsulation. Future studies should therefore quantify anthocyanin retention (e.g., total anthocyanins or HPLC profiling at 0, 7, 14, and 21 days of storage) to validate the protective role of nanoencapsulation against microbial metabolism and oxidative degradation.

### 2.7. Sensory Analysis

According to the sensory analysis data (Table 3), formulation Y-C tends to receive higher scores in most sensory attributes compared to anthocyanin-enriched formulations (Y-Ant-5 and Y-Ant-10). Specifically, in attributes such as flavor, aftertaste, aroma, consistency, texture, appearance, and overall acceptance, formulation Y-C obtains higher rating values compared to Y-Ant-5 and Y-Ant-10. This suggests that the nano-encapsulation of anthocyanins may affect the sensory perception of yogurt, including its flavor, aroma, texture, and appearance. However, statistical analysis indicates that there are no significant differences between the results of some attributes. Regarding overall acceptance, the values remained statistically nonsignificant. Therefore, all three formulations were found to have the same acceptance.

Anthocyanins may subtly mediate the flavor, as their characteristic taste varies depending on the specific source. When anthocyanins are added to yogurt, they may impart a slight fruity or berry flavor, as many natural sources of anthocyanins come from fruits such as blackberries, blueberries, cherries, raspberries, and Açaí [82,83]. This fruity or berry flavor may be perceptible in yogurt, especially in formulations with higher concentrations of anthocyanins. However, the intensity of the flavor will depend on various factors, such as the anthocyanin concentration added, the source of the anthocyanins, and the presence of other ingredients that may influence the yogurt’s flavor; hence, they must be considered in formulating to ensure that the final result is pleasing to consumers [15,83]. Finally, in terms of consumer acceptability, formulation Y-C also appears to be preferred, as it receives a slightly higher score in overall acceptance compared to Y-Ant-5 and Y-Ant-10.

Matos et al. [84] produced a yogurt enriched with *Isochrysis galbana*, which offers significant nutritional benefits, such as increasing ω3 long-chain polyunsaturated fatty acids (LC-PUFAs), particularly DHA, and improving the ω3/ω6 ratio, which is beneficial for cardiovascular and neurological health. However, in vitro digestion studies indicate low bioaccessibility of DHA and EPA, suggesting limited availability of these essential fatty acids during digestion. Sensory acceptability, including green coloration and possible fishy taste, needs further evaluation through consumer testing and sensory panels. With the growing interest in functional foods, this yogurt has significant potential in the health-conscious consumer market, although additional studies are required to confirm bioavailability and optimize the formulation. Therefore, yogurt enriched with nanoliposomes loaded with anthocyanins, considering the physicochemical and rheological analyses as well as sensory attributes, could have greater commercial acceptability among consumers.

## 3. Materials and Methods

### 3.1. Ethical Handling of Human Erythrocyte Membrane-Based Assays

All procedures involving human red blood cells (RBCs) were performed in adherence to international standards, including the FDA regulations outlined in the Code of Federal Regulations (Title 21, Part 640, Subpart B—Red Blood Cells, Section 640.14), as well as the Official Mexican Standard NOM-253-SSA1-2012 [85], which governs the collection, processing, and use of blood and its components for therapeutic use. The erythrocyte membranes utilized in this study were provided by the clinical analysis laboratory at the University of Guadalajara, which holds accreditation under ISO/IEC 17025 (NMX-EC-17025) and ISO 15189, issued by the ISO/TC 212 committee (focused on clinical laboratory testing and in vitro diagnostic systems), using ISO/IEC 17025 and ISO 9001 as reference frameworks [86,87,88,89]. Blood samples were obtained from healthy adult donors, aged between 20 and 40 years, with an erythrocyte count ranging from approximately 4.7 to 6.1 × 10^6^ cells/µL. Each participant provided written informed consent before sample collection. Venous blood was drawn using a sterile collection tube containing EDTA as an anticoagulant. The isolated erythrocyte membranes served as the experimental model to assess the erythroprotective activity of the yogurt formulation [90,91,92]. The study was conducted following the institution’s approved protocol (CI 2023-47).

### 3.2. Extraction of Anthocyanins by High-Energy Ultrasound

Anthocyanins were obtained as a food-grade lyophilized powder of *Euterpe oleracea* (açaí) supplied by Hunan World Well-Being Bio-Tech Co., Ltd. (Changsha, China), a manufacturer certified with ISO and FDA standards offering standardized açaí extracts with different anthocyanin contents. The extract was processed using a high-energy ultrasound-assisted method in physiological solution (0.9% NaCl). Briefly, 1 g of dried and ground açaí sample was suspended in 20 mL of physiological solution and subjected to ultrasonic treatment using a probe-type ultrasonic processor (VCX-130, Sonics & Materials Inc., Newtown, CT, USA; maximum output power 130 W, frequency 20 kHz). The sonication was applied at a 55% amplitude in 60 s on/off cycles for 15 min, while maintaining the suspension in an ice bath to prevent thermal degradation. The extract was subsequently centrifuged at 10,000 rpm for 10 min at 4 °C, and the supernatant containing the anthocyanins was collected, filtered through a 0.22 µm membrane, and stored at −20 °C until further analysis.

### 3.3. Antioxidant Properties of Food-Grade Anthocyanins

The antioxidant activity of food-grade anthocyanins was assessed using DPPH [37], ABTS [38], and FRAP [39] assays, employing a microplate reader to quantify the free radical scavenging or reducing capacity. In the DPPH assay, an ethanolic solution was prepared and adjusted to an absorbance of 0.7 ± 0.02 at 515 nm; subsequently, 20 µL of the sample was mixed with 200 µL of the DPPH solution and allowed to react for 30 min in the dark before absorbance measurement. For the ABTS assay, the ABTS^+^• cation radical was generated after 16 h of incubation with potassium persulfate, followed by dilution with ethanol to reach an absorbance of 0.7 ± 0.02 at 734 nm. Then, 20 µL of the sample was added to 270 µL of the ABTS^+^• solution, and the mixture was incubated in the dark for 30 min prior to reading. Regarding the FRAP assay, a working solution was prepared by mixing sodium acetate buffer, TPTZ, and ferric chloride in a 10:1:1 ratio. Then, 20 µL of the sample was combined with 280 µL of the FRAP solution and incubated for 30 min in the dark. The absorbance was measured at 638 nm. All determinations were performed in triplicate, and the results were expressed as percentage inhibition (DPPH and ABTS) and as µmol Trolox equivalents per gram of dry weight (FRAP).

### 3.4. Erythroprotective Potential

#### 3.4.1. Protective Effect Against AAPH-Induced Oxidative Hemolysis

The antihemolytic activity of food-grade anthocyanins was assessed by inducing oxidative hemolysis using the free radical generator AAPH [2,2′-azobis(2-methylpropionamidine)], based on the methodologies described by Ruiz-Cruz et al. [91]. Erythrocytes were isolated from healthy adult volunteers aged 20–45 years after obtaining informed consent. Samples included erythrocytes from O+ and O− blood groups to evaluate erythroprotective activity in preliminary personalized nutrition assessments. A 2% erythrocyte suspension and a 40 mM AAPH solution (pH 7.4) were prepared. The assay involved mixing 100 μL of erythrocyte suspension, 100 μL of sample, and 100 μL of AAPH solution. The positive control consisted of erythrocytes mixed with PBS and AAPH, while the negative control consisted of erythrocytes mixed with PBS only. All samples and controls were incubated at 37 °C for 3 h with constant agitation at 45 rpm. Following incubation, 1 mL of PBS was added to each sample, which was then centrifuged at 1500 rpm for 10 min. Hemoglobin release due to oxidative damage was measured at 540 nm using a 96-well microplate reader.

Morphological alterations in erythrocytes exposed to AAPH-induced oxidative stress were examined using optical microscopy (Eclipse FN1 microscope). Immediately after spectrophotometric analysis, a blood smear was prepared using the supernatant obtained from the hemolysis reaction and stained with Wright’s stain to visualize damaged erythrocytes. Micrographs were captured at 100× magnification using the NIS-Elements F imaging software, Version 4.60 (Nikon Corporation, Tokyo, Japan, accessed on 1 March 2025) and scale bars of 5 μm were included for size comparison.

#### 3.4.2. Photoprotective Effect Against UV Radiation-Induced Oxidative Hemolysis

The photoprotective activity of food-grade anthocyanins was evaluated through UV-induced hemolysis in human erythrocytes [13]. Cells from various ABO and Rh blood groups were irradiated with UV-A (315–395 nm) and UV-B (280–315 nm) light using a horizontal setup with two UV lamps (0.85 mW/cm^2^) at a controlled temperature of 18 ± 1 °C. Anthocyanins, dissolved in PBS (0.5 M), were tested for their protective effect. A 1% erythrocyte suspension was prepared, and 3 mL aliquots were placed in sterile tubes, followed by the addition of 150 µL of the sample. Positive controls consisted of irradiated erythrocytes without treatment, while non-irradiated cells served as negative controls. Samples were pre-incubated at 37 °C for 30 min, then exposed to UV-A and UV-B for 0, 15, 30, 60, and 120 min at a 10 cm distance. After exposure, samples were centrifuged at 2000× *g* for 10 min, and 300 µL of the supernatant was transferred to a 96-well plate. Hemolysis was quantified by measuring absorbance at 540 nm.

### 3.5. Synthesis and Physicochemical Characterization of Anthocyanin-Loaded Nanoliposomes

#### 3.5.1. Synthesis of Anthocyanin-Loaded Nanoliposomes

Anthocyanin-loaded nanoliposomes (ANT-LN) were prepared using the ultrasonic film dispersion method. Briefly, non-hydrogenated soy phosphatidylcholine (SPC, 70 mg) and cholesterol (14 mg) were dissolved in 20 mL of chloroform–methanol (2:1 *v*/*v*) in a round-bottom flask at a 5:1 *w*/*w* ratio. The dried lipid film was hydrated with an anthocyanin solution (1 mg/mL, phosphate buffer, pH 5.0), followed by probe sonication using an ultrasonic processor (VCX-130, Sonics & Materials Inc., Newtown, CT, USA; maximum output power 130 W, frequency 20 kHz). The sonication was applied at 55% amplitude in 60 s on/off cycles for 15 min, with the suspension kept in an ice bath to prevent overheating and anthocyanin degradation. The organic phase was evaporated under reduced pressure at 40–50 °C using a rotary evaporator (Heidolph Laborata 4000, Germany; Buchi R-210, BÜCHI Labortechnik AG, Flawil, Switzerland) until a thin, uniform lipid film was formed on the flask wall. To ensure complete solvent removal, the lipid film was further dried under nitrogen flow for 1 h.

The dried lipid film was hydrated with 10 mL of anthocyanin solution (1 mg/mL, equivalent to 10 mg of total anthocyanins) prepared in phosphate buffer (0.05 mol/L, pH 5.0). This corresponds to a lipid:bioactive ratio of approximately 10:1 (*w*/*w*). Hydration was performed at 40–50 °C with continuous stirring for 30 min to promote the spontaneous formation of multilamellar vesicles (MLVs).

To reduce particle size and obtain small unilamellar vesicles (SUVs), the suspension was subjected to high-energy sonication using a probe-type ultrasonic processor (Branson Digital Sonifier, Qsonica, USA; VCX-130, Sonics, Danbury, CT, USA) at 55% amplitude in 60 s on/off cycles for 15 min, with the sample maintained in an ice bath to prevent overheating and anthocyanin degradation. The suspension was then centrifuged at 10,000 rpm for 10 min at 4 °C to remove unencapsulated anthocyanins. The resulting nanoliposomes exhibited a particle size below 200 nm, high encapsulation efficiency, and improved stability. The final ANT-LN suspension was stored at 4 °C in amber glass vials until further use.

As part of the methodology, analyses performed directly on nanoliposomes included encapsulation efficiency, particle size, polydispersity index, zeta potential, aqueous dispersibility, in vitro release, and kinetic modeling (Korsmeyer–Peppas). In contrast, scanning electron microscopy (SEM) was conducted on lyophilized yogurt enriched with anthocyanin-loaded nanoliposomes to evaluate their structural integration within the matrix.

#### 3.5.2. Encapsulation Efficiency

Encapsulation efficiency was determined using a solvent extraction method adapted from Pan et al. [40]. Briefly, 400 μL of the ANT-LN suspension was mixed with 1 mL of petroleum ether and agitated at 45 rpm for 5 min at 30 °C. The mixture was then centrifuged at 3000 rpm for 5 min to separate the phases. The upper layer was collected and subjected to rotary evaporation to eliminate residual solvent. The remaining extract was dissolved in chloroform, and unencapsulated anthocyanins were quantified at 460 nm using a UV-Vis microplate reader (Multiskan GO, (Multiskan GO, Thermo Fisher Scientific, Vantaa, Finland). Encapsulation efficiency (%) was calculated using the following equation: Encapsulation efficiency (EE %) of anthocyanin-loaded nanoliposomes (ANT-LN) was determined using a separation method adapted from previous studies with modifications to ensure accuracy. Briefly, 400 μL of the ANT-LN suspension was placed in a centrifuge tube and mixed with 1 mL of acidified ethanol (ethanol containing 0.1% HCl *v*/*v*), a solvent capable of efficiently solubilizing free anthocyanins without disrupting the liposomal bilayer. The mixture was vortexed for 2 min and centrifuged at 12,000 rpm for 15 min at 4 °C to separate the free anthocyanins (supernatant) from the encapsulated fraction (pellet).

The supernatant containing free anthocyanins was collected, and absorbance was measured at 530 nm, corresponding to the maximum visible absorption of anthocyanins in acidic medium, using a UV-Vis microplate reader (Multiskan GO, Thermo Fisher Scientific, USA). The total anthocyanin content (TAC) of the liposomal dispersion was determined after complete disruption of nanoliposomes by addition of 1% Triton X-100 (*v*/*v*) in acidified ethanol, followed by quantification at 530 nm.

(1) $$Encapsulation\;Efficiency\;(\%)=\frac{TAC-FC}{TAC\;}\times 100$$

where *TAC* is the total anthocyanin concentration after liposome disruption, and *FC* is the free anthocyanin concentration quantified in the supernatant.

#### 3.5.3. Assessment of Particle Size and Zeta Potential Stability

An aliquot of liposomal dispersion (1.0 mL) was diluted in 100 mL of phosphate buffer and then transferred into polystyrene cuvettes for analysis. The hydrodynamic diameter (Dz) and zeta potential (ζ) were measured using a Nano-ZS90 particle size analyzer (Malvern Instruments Ltd., Malvern, UK). All determinations were carried out in triplicate to ensure the reliability of the results.

#### 3.5.4. Aqueous Dispersibility Assay

The aqueous dispersibility of anthocyanin-loaded nanoliposomes (ANT-LN) was determined following a procedure adapted from Pan et al. [40], with modifications to ensure selectivity for anthocyanins. Briefly, an aliquot of the ANT-LN suspension equivalent to 1 mg of formulation was dispersed in 1 mL of distilled water under gentle stirring for 10 min at room temperature. The mixture was then centrifuged at 12,000 rpm for 15 min at 4 °C to separate insoluble lipid residues from the soluble fraction.

The supernatant was collected, and the anthocyanin concentration was quantified at 530 nm using a UV-Vis microplate reader (Multiskan GO, (Multiskan GO, Thermo Fisher Scientific, Vantaa, Finland) under acidic conditions (ethanol–HCl 0.1% *v*/*v*) to ensure stability of the chromophore. The dispersibility of ANT-LN was calculated as the anthocyanin concentration (μg/mL) present in the aqueous phase, normalized according to the anthocyanin content determined in the encapsulation efficiency assay.

#### 3.5.5. In Vitro Release

In vitro release testing of ANT-LN was performed by placing 10 mL of the nanoliposome suspension, containing 1 mg of anthocyanins, into a dialysis bag with a molecular weight cutoff of 8000–14,000 Da. The sealed bag was submerged in 100 mL of phosphate-buffered saline (PBS, 0.05 mol/L, pH 7.4) and maintained at 37 °C under continuous stirring at 100 rpm. At predetermined time intervals (0.5, 1, 2, 4, 6, 8, 10, 12, and 24 h), 1 mL aliquots were collected from the external medium. The amount of anthocyanin released was quantified by measuring the absorbance at 460 nm using a UV-Vis microplate reader. All experiments were conducted in triplicate [56], and the release profile was expressed as the percentage of anthocyanins released over time using the following equation:

(2) $$Release\;rate=\;\frac{Released}{{Ant}_{total}\;}\;\times \;100$$

In vitro release data were obtained at predetermined time intervals (0.5 to 24 h), and the cumulative release percentage (Mt/M∞) was calculated. The data were transformed to a log-log scale (log Mt/M∞ vs. log t) and fitted to the Korsmeyer–Peppas equation to determine the release exponent (n) and kinetic constant (k). Linear regression was applied to obtain model parameters, and the coefficient of determination (R^2^) was calculated to assess the goodness of fit. All experiments were performed in triplicate, and results were reported as mean ± standard deviation. The data were fitted to the model equation:

(3) $$\frac{M_{t}}{M_{\infty }}=k\times t^{n}$$

### 3.6. Development of Functional Yogurt Enriched with Anthocyanin-Loaded Nanoliposomes

The methodological approach for the formulation and evaluation of yogurt enriched with anthocyanin-loaded nanoliposomes is summarized into four sequential phases: (i) preparation of the artisanal yogurt base; (ii) enrichment of the yogurt with the nanoliposomes; (iii) refrigerated storage under controlled conditions; and (iv) comprehensive multiparametric analyses to assess the effects of enrichment on key physicochemical, functional, and structural properties of the product.

#### 3.6.1. Preparation and Formulation of Artisanal Yogurt

Artisanal yogurt was sourced from a certified local producer in Ameca, Jalisco, Mexico, which manufactures small batches under traditional practices, thereby justifying the use of the term “artisanal.” The process began with raw cow’s milk, which was subjected to pasteurization at 80 °C for 30 min to ensure safety and compliance with NOM-243-SSA1-2010. Pasteurized milk was then inoculated with the classical starter cultures *Lactobacillus delbrueckii* subsp. *bulgaricus* and *Streptococcus thermophilus*, recognized globally as the essential microorganisms for yogurt fermentation in accordance with international quality standards [19]. These bacteria play a critical role in acidifying the medium, developing the final texture, and enhancing the sensory characteristics of yogurt, while also providing potential probiotic benefits.

**Figure 14:**
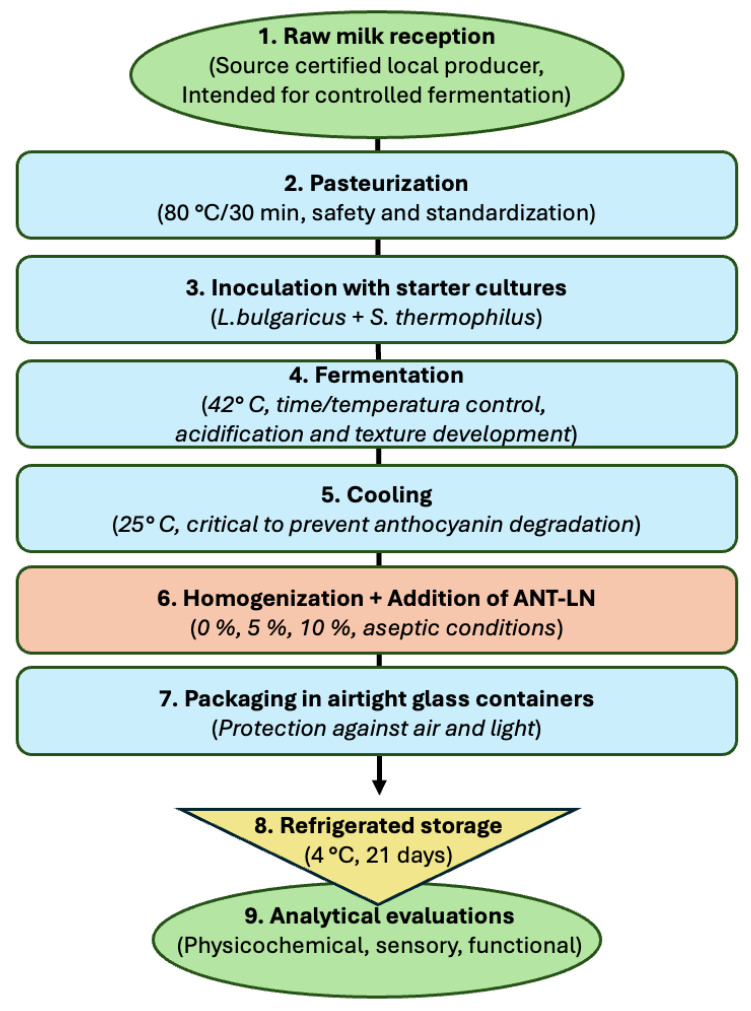
Flow diagram illustrating the preparation of artisanal yogurt enriched with anthocyanin-loaded nanoliposomes (ANT-LN). The process includes (1) raw milk reception, (2) pasteurization, (3) inoculation with starter cultures, (4) fermentation, (5) cooling, (6) homogenization and addition of ANT-LN, (7) packaging, (8) refrigerated storage, and (9) analytical evaluations.

To ensure uniformity in fermentation parameters (time, temperature, and inoculum ratios), the research team directly supervised the production process. This guaranteed methodological reproducibility prior to the enrichment step with anthocyanin-loaded nanoliposomes (ANT-LN). After fermentation was completed and the yogurt cooled to 25 °C, anthocyanin-loaded nanoliposomes were incorporated during the homogenization step under aseptic conditions, ensuring even distribution and minimizing anthocyanin degradation. The workflow of the process, from raw milk to yogurt enrichment, is summarized in Figure 14 (flow diagram).

Anthocyanin-loaded nanoliposomes were prepared using the ultrasonic film dispersion technique and incorporated into artisanal yogurt at concentrations of 0%, 5%, and 10% (*w*/*w*) in separate 100 g samples, resulting in the formulations Y-C (control yogurt), Y-ANT-5, and Y-ANT-10. Each formulation was homogenized and placed in airtight glass containers, stored under refrigeration at 4 °C, and protected from exposure to air and light until further analysis.

The experimental workflow involved the following steps: (1) synthesis of nanoliposomes from phosphatidylcholine, cholesterol, and anthocyanins; (2) yogurt fermentation; (3) incorporation of nanoliposomes during homogenization; (4) sensory evaluation prior to storage; (5) refrigerated storage for 21 days; (6) monitoring of pH, titratable acidity, electrical conductivity, syneresis, and Aqueous Dispersibility Assay during storage; and (7) evaluation of stability and functional properties throughout the storage period.

#### 3.6.2. Morphological Evaluation of Lyophilized Yogurt Enriched with Nanoliposomes

A morphological analysis was conducted to assess the integrity and stability of anthocyanin-loaded nanoliposomes (Ant-LN) after their incorporation into the yogurt matrix [55]. Lyophilized samples of enriched yogurt were prepared to evaluate the interaction between the nanocarriers and the food matrix. The aim was to verify whether the nanoliposomes preserved their morphology, size, and structure within the dairy system. Samples were mounted on copper tape, coated with gold, and analyzed using scanning electron microscopy (SEM, JSM-6610LV, JEOL Ltd., Tokyo, Japan).This technique confirmed the nanoliposomes’ stability and distribution, supporting their structural preservation and potential functionality post-incorporation [15,19].

### 3.7. Chemical Characterization

The chemical composition of both the control (artisanal yogurt) and the yogurt enriched with nanoliposomes containing anthocyanin was analyzed to determine levels of dry matter, moisture, proteins, lipids, ash, and carbohydrates. These proximate analyses were carried out in accordance with the procedures described in AOAC Method 925.23 (Association of Official Analytical Chemists) [93]. All measurements were performed in triplicate for each formulation to ensure accuracy and reproducibility.

### 3.8. Microbiological Assessment of Yogurt

To verify the hygienic and safety standards of the yogurt prior to sensory testing, microbiological evaluations were carried out. The analyses included quantification of total aerobic mesophilic bacteria, total and fecal coliforms, and molds and yeasts, in accordance with the specifications outlined in NOM-243-SSA1-2010, which regulates fermented milk products. For aerobic mesophilic counts, serial dilutions of yogurt samples (1 mL in 9 mL sterile peptone water) were prepared and plated on Plate Count Agar (PCA, Merck KGaA, Darmstadt, Germany), followed by incubation at 35 ± 2 °C for 48 h. Total coliforms were enumerated on Violet Red Bile Agar (VRBA, Merck) after incubation at 37 ± 1 °C for 24 h, while fecal coliforms were confirmed by incubation at 44.5 °C for 24 h using the same medium. Molds and yeasts were quantified on Sabouraud Dextrose Agar (SDA, Merck) incubated at 25 ± 2 °C for 5 days. All microbiological determinations were performed in triplicate for each yogurt formulation (Y-C, Y-ANT-5, and Y-ANT-10) to ensure reproducibility. The results were compared with the microbiological safety criteria established for dairy products in NOM-243-SSA1-2010. All formulations complied with the permissible limits, thereby confirming their microbiological safety and suitability for subsequent sensory evaluation [94].

It should be noted that specific enumeration of lactic acid bacteria (LAB) was not included in this study, as the microbiological assessment focused on general safety and hygienic quality indicators in compliance with NOM-243-SSA1-2010. The primary objective was to ensure product safety prior to sensory evaluation, while the main emphasis of this research was on anthocyanin stability and nanoencapsulation performance within yogurt.

### 3.9. Stability of ANT-LN Enriched Yogurt Under Cold Storage: Physicochemical and Rheological Insights

#### 3.9.1. Determination of Physicochemical Properties

The physicochemical characterization of yogurt samples included the evaluation of pH [95,96], titratable acidity [95,96], syneresis susceptibility (STS) [97], and water absorption capacity (WAC) [89,98]. The pH was determined using a calibrated digital pH meter (HI 2211 PH/MV, HANNA Instruments, Woonsocket, RI, USA) after diluting 1 g of sample in 10 mL of distilled water, in accordance with AOAC (2012) guidelines. Titratable acidity was assessed following the AOAC method 942.15 (2000), by mixing 10 g of yogurt with 10 mL of hot distilled water and titrating with 0.1 N NaOH using phenolphthalein as an indicator; the results are expressed as grams of lactic acid per 100 g of product. Syneresis was determined by centrifuging 20 g of yogurt at 500 rpm for 5 min and quantifying the separated whey to calculate the percentage loss, according to Achanta et al. [97]. For WAC determination, 2.5 g of sample was mixed with 30 mL of distilled water, incubated at 30 °C for 30 min, and centrifuged at 3000× *g* for another 30 min. The supernatant was discarded, and the remaining pellet was dried at 90 °C for 24 h and then weighed to calculate WAC as a percentage of water holding capacity.

#### 3.9.2. Texture

The texture of the yogurt was assessed using the TA-XT2 texture analyzer (Stable Micro Systems, Godalming, UK) equipped with software [99]. The probe and conditions were a cylindrical probe diameter of 25 mm (P25/L), pre-test speed = 5 mm/s, test speed of 3.0 mm/s, target mode = strain, time of 3 s, and trigger force = 0.5 g with 2000 g calibration weight. The test was performed directly on a 40 g sample cup, conducted in triplicate. All experiments were conducted at 5 °C. The key textural characteristics of the set yogurt were tested, including firmness or gel strength (peak compression force during penetration) and adhesiveness (negative force area).

To evaluate the structural degradation of yogurt formulations over time, a first-order kinetic model was applied to the experimental data of firmness and consistency during 21 days of refrigerated storage. The first-order model assumes that the rate of texture loss is proportional to its current value, describing an exponential decay over time. Nonlinear regression analysis was performed using the least squares method to fit the experimental data and obtain the degradation rate constant (k). Model fitting and curve plotting were carried out using Python version 3.11.5 and the curve_fit function from SciPy version 1.11.3 (accessed on 28 March 2025).

The mathematical expression of the first-order kinetic model is as follows:

(4) $$T\left(t\right)=T_{0}e^{-kt}$$

where T(t) is the firmness or consistency at time *t*; T_0_ is the initial value at day 0; *k* is the degradation rate constant (day^−1^); *t* is the storage time (days). This model enables comparison of the textural stability among formulations and supports shelf-life prediction and formulation optimization.

#### 3.9.3. Rheological Analysis

Measurements were conducted using a rotational viscometer (Thermo Haake DC 10, model VT 550, Karlsruhe, Germany), with concentric cylinders (NV ST 807-0713 CE and NV 807-0702, Thermo Haake GmbH, Karlsruhe, Germany), and the data were collected with the Pro Rheowin software program © (version 2.93, Haake) [48]. These analyses were performed in triplicate. The temperature used was 25 °C, and the shear rate ranged from 0 to 2000 s^−1^ (upward curve) and from 2000 to 0 s^−1^ (downward curve); each curve was obtained over 3 min. The flow behavior was described using the Power Law model, and the thixotropic behavior of the yogurt was evaluated by calculating the area of the hysteresis loop between the upward and downward flow curves.

### 3.10. Sensory Analysis

The sensory evaluation procedure was conducted following the principles outlined by Lawless et al. [98] and the methodology described by Stone and Sidel [100]. Twenty trained panelists aged between 25 and 35 years were selected to participate in the sensory panel. The sensory evaluation procedure was conducted following the principles outlined by Lawless and Heymann [98] and the methodology described by Stone and Sidel [100]. Twenty trained panelists aged between 25 and 35 years were selected to participate in the sensory panel. The evaluation considered the attributes of color, flavor, aftertaste, scent, consistency, texture, appearance, and overall acceptance. A 9-point hedonic scale was employed to assess acceptability, where 0 indicated very unpleasant and 9 indicated very pleasant. Panelists were instructed to assign a numerical value that best described the intensity of their perception for each attribute, and samples were arranged in ascending order according to preference. Three yogurt formulations were evaluated: Y1 (control yogurt, Y-C), Y2 (yogurt enriched with 5% ANT-LN, Y-ANT-5), and Y3 (yogurt enriched with 10% ANT-LN, Y-ANT-10). All evaluations were performed under controlled environmental conditions with standardized sample presentation to minimize bias and ensure reproducibility of the results [98].

### 3.11. Statistical Analysis

The data are presented as mean values accompanied by their corresponding standard deviations (SDs), based on a minimum of three independent replicates (n > 3). Statistical analyses were conducted using JMP software version 16 for Mac. Differences among groups were evaluated through one-way and two-way analysis of variance (ANOVA) to assess the influence and interaction of the studied factors. Tukey’s post hoc test was applied, considering a significance threshold of *p* < 0.05 [19].

## 4. Conclusions

The present study demonstrates an innovative approach to functional food development through the incorporation of anthocyanin-loaded nanoliposomes into artisanal yogurt. This research introduces a novel delivery system that enhances the stability, bioavailability, and controlled release of anthocyanins, bioactive compounds known for their antioxidant, erythroprotective, and neuroprotective properties. The process of nanoencapsulation successfully protected anthocyanins from degradation and allowed a gradual release, confirmed by in vitro kinetics modeled with Korsmeyer–Peppas, indicating a non-Fickian mechanism. The encapsulation efficiency reached over 91%, while the high water solubility and retention stability under stress conditions ensured practical application. Importantly, yogurts enriched with these nanoliposomes showed reduced syneresis, enhanced water-holding capacity, and acceptable pH and titratable acidity over 21 days of cold storage. Though the viscosity and texture declined slightly with enrichment, the formulations remained within acceptable sensory thresholds, achieving comparable consumer acceptance. Furthermore, enriched yogurts maintained microbiological safety and demonstrated improved physicochemical resilience. These results not only validate the technological feasibility of nanoliposome incorporation in dairy matrices but also highlight their potential as carriers for neuronutrient in functional foods. This opens avenues for designing targeted nutritional interventions and biotargeted products aimed at populations susceptible to oxidative stress-related conditions.

## Figures and Tables

**Table 1:** Physicochemical characterization of anthocyanin-loaded nanoliposomes. Data represent the mean value ± standard deviation (n = 3).

Formulation	EE (%)	Particle Size (nm)	Polydispersity Index (PDI)	Zeta Potential (mV)	Interpretation
ANT-LN	91.34 ± 1.34	132 ± 14.23	0.21 ± 0.031	−34.61 ± 1.82	Small, homogeneous vesicles with high EE and strong electrostatic stability, optimal for functional applications.

**Table 2:** Proximate Composition of Yogurt Enriched with Anthocyanin-Loaded Nanoliposomes.

			Total Dry Weight			
Samples	Dry Matter (%)	Moisture (%)	Protein (%)	Fat (%)	Carbohydrates (%)	Ashes (%)
Y-C	11.6 ± 1.3 ^b^	88.4 ± 1.5 ^a^	35.1 ± 2.8 ^a^	32.4 ± 2.5 ^a^	26.0 ± 1.5 ^a^	6.5 ± 0.7 ^a^
Y-ANT-5	15.8 ± 1.32 ^a^	84.2 ± 1.6 ^b^	37.2 ± 2.3 ^a^	33.8 ± 2.3 ^a^	21.9 ± 1.7 ^b^	7.1 ± 0.5 ^a^
Y-ANT-10	12.2 ± 2.75 ^ab^	87.8 ± 1.3 ^ab^	34.4 ± 1.53 ^a^	33.6 ± 2.1 ^a^	25.2 ± 2.5 ^ab^	6.8 ± 0.8 ^a^

The values represent mean averages. Different superscript letters indicate statistically significant differences between samples according to the post hoc test (one-way ANOVA, *p* > 0.05). Data are presented as the mean of three replicates. Mean ± standard deviation (SD) of n = 3. Y-C = Control yogurt without nanoliposome; Y-ANT-5 = Yogurt enriched with 5% anthocyanin-loaded nanoliposome; Y-ANT-10 = Yogurt enriched with 10% anthocyanin-loaded nanoliposome.

**Table 3:** Effect of anthocyanin-loaded nanoencapsulation on the sensory characteristics of yogurt enriched with anthocyanins. In the Figure, lowercase letters indicate the sensory scale ranging from 0 (very unpleasant) to 9 (very pleasant).

**Sensory Quality Attributes**	**Treatments**			** 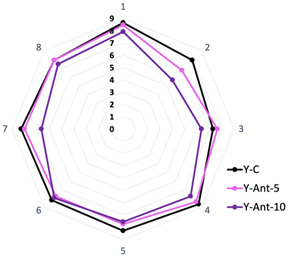 **
	**Y-C**	**Y-Ant-5**	**Y-Ant-10**	
Color	8.64 ± 0.16 ^a^	8.45 ± 0.26 ^a^	7.91 ± 0.62 ^a^	
Flavor	7.91 ± 0.23 ^a^	6.73 ± 0.42 ^b^	5.64 ± 0.34 ^c^	
Aftertaste	7.27 ± 0.26 ^b^	7.64 ± 0.25 ^a^	6.34 ± 0.73 ^a^	
Scent	8.64 ± 0.45 ^a^	8.36 ± 0.45 ^ab^	7.73 ± 0.23 ^b^	
Consistency	8.27 ± 0.35 ^a^	7.73 ± 0.34 ^ab^	7.55 ± 0.34 ^b^	
Texture	8.18 ± 0.23 ^a^	7.73 ± 0.65 ^a^	7.91 ± 0.45 ^a^	
Appearance	8.27 ± 0.32 ^a^	8.00 ± 0.32 ^a^	6.64 ± 0.34 ^b^	
General acceptance	7.91 ± 0.15 ^a^	7.85 ± 0.21 ^a^	7.45 ± 0.23 ^a^	

## Data Availability

The information that underpins the results of this study is included in the main body of the article. Further specific data or clarifications are available from the corresponding authors upon reasonable request.
